# Targeting antimalarial metabolites from the actinomycetes associated with the Red Sea sponge *Callyspongia siphonella* using a metabolomic method

**DOI:** 10.1186/s12866-023-03094-3

**Published:** 2023-12-12

**Authors:** Noha M. Gamaleldin, Hebatallah S. Bahr, Natalie Millán-Aguiñaga, Mahshid Danesh, Eman M. Othman, Thomas Dandekar, Hossam M. Hassan, Usama Ramadan Abdelmohsen

**Affiliations:** 1 Department of Microbiology, Faculty of Pharmacy, the British University in Egypt (BUE), Cairo, 11837 Egypt; 2 Department of Pharmacognosy, Faculty of Pharmacy, Nahda University, Beni-Suef, Egypt; 3 Facultad de Ciencias Marinas, Universidad Autónoma de Baja California, Ensenada, 22860 Baja California México; 4 Department of Bioinformatics, University of Würzburg, Am Hubland, 97074 BiocenterWürzburg, Germany; 5 Department of Biochemistry, Faculty of Pharmacy, Minia University, Minia, 61519 Egypt; 6 Department of Pharmacognosy, Faculty of Pharmacy, Beni-Suef University, Beni-Suef, Egypt; 7 Department of pharmacognosy, faculty of Pharmacy, Minia University, Minia, Egypt; 8 Department of pharmacognosy, faculty of Pharmacy, Deraya University, New Minia City, 61111 Minia Egypt

**Keywords:** *Callyspongia siphonella*, *Streptomyces*, PCA, PLS-DA, Antimalarial, Metabolomics, Quinones

## Abstract

Malaria is a persistent illness that is still a public health issue. On the other hand, marine organisms are considered a rich source of anti‑infective drugs and other medically significant compounds. Herein, we reported the isolation of the actinomycete associated with the Red Sea sponge *Callyspongia siphonella*. Using "one strain many compounds" (OSMAC) approach, a suitable strain was identified and then sub-cultured in three different media (M1, ISP2 and OLIGO). The extracts were evaluated for their in-vitro antimalarial activity against *Plasmodium falciparum* strain and subsequently analyzed by Liquid chromatography coupled with high-resolution mass spectrometry (LC-HR-MS). In addition, MetaboAnalyst 5.0 was used to statistically analyze the LC-MS data. Finally, Molecular docking was carried out for the dereplicated metabolites against lysyl-tRNA synthetase (PfKRS1)*.* The phylogenetic study of the 16S rRNA sequence of the actinomycete isolate revealed its affiliation to *Streptomyces* genus. Antimalarial screening revealed that ISP2 media is the most active against *Plasmodium falciparum* strain. Based on LC-HR-MS based metabolomics and multivariate analyses, the static cultures of the media, ISP2 (ISP2-S) and M1 (M1-S)**,** are the optimal media for metabolites production. OPLS-DA suggested that quinone derivatives are abundant in the extracts with the highest antimalarial activity. Fifteen compounds were identified where eight of these metabolites were correlated to the observed antimalarial activity of the active extracts. According to molecular docking experiments, saframycin Y3 and juglomycin E showed the greatest binding energy scores (-6.2 and -5.13) to lysyl-tRNA synthetase (PfKRS1), respectively. Using metabolomics and molecular docking investigation, the quinones, saframycin Y3 (5) and juglomycin E (1) were identified as promising antimalarial therapeutic candidates. Our approach can be used as a first evaluation stage in natural product drug development, facilitating the separation of chosen metabolites, particularly biologically active ones.

**Supplementary Information:**

The online version contains supplementary material available at 10.1186/s12866-023-03094-3.

## Introduction

Malaria is a long-standing disease and remains a public health problem that most likely originating in Africa [1]. It affects 30 million people globally and kills about 2.5 million people each year, mostly children under the age of five. Chloroquine-resistant *Plasmodium falciparum* strains are now ubiquitous in all endemic locations around the world [2], and there is also evidence of the emergence of resistance against artemisinin-based combination therapies, which now serve as first line drugs for the treatment of *P. falciparum* [3]. Therefore, there is a growing need to identify new drugs. Marine ecosystems are composed of taxonomically and biologically diverse macro- and microorganisms that can withstand pressure, salinity, and temperature extremes. They are capable of producing unique compounds with novel therapeutic applications that aren't found in their terrestrial equivalents [4]. Sponges (phylum Porifera) are among the oldest and most abundant multicellular marine organisms. The Red Sea sponge, *Callyspongia siphonella* (family Callyspongiidae) is found in shallow water throughout the Gulfs of Aqaba and Suez mostly deeper than 5 m [5]. On the other hand, microbial populations, including archaea, bacteria, fungus and viruses abound in the interiors of different sponge species [6]. So far, at least 32 bacterial phyla and candidate groups have been identified in marine sponges, including Acidobacteria, Actinobacteria, Chloroflexi, Nitrospira, Cyanobacteria, Bacteriodetes, Gemmatimonadetes, Planctomycetes, Spirochaetes and Proteobacteria. Actinomycetes, particularly those associated with marine sponges are well-known for their capacity to generate novel lead molecules including phenazines, peptides and alkaloids of clinical and pharmacological significance, such as: antibacterial, antifungal, antiparasitic, immunomodulatory, anti-inflammatory, antioxidant, and anticancer activities [6–8]. However, many secondary metabolites encoded in actinomycete genomes are yet to be found, owing to the fact that these genes are not transcribed under standard laboratory conditions. The "one strain many compounds" (OSMAC) strategy involves manipulating fermentation conditions and is an efficient way to activate metabolic pathways that are either silent or poorly expressed [9]. In the present study, the actinomycete *Streptomyces* associated with the Red Sea sponge *C. siphonella* was isolated, identified and cultured in various culture media utilizing the OSMAC approach to determine the optimal media for the production of beneficial secondary metabolites. Antimalarial screening was performed to assess the effectiveness of *Streptomyces* species fermented extracts against *P. falciparum* strain. LC-HR-MS assisted chemical investigation followed by multivariate data statistical analysis (MVDA) was carried out in order to reduce the amount of data collected and find correlation and differentiation among the samples tested. Finally, molecular docking analysis was performed for the suggested compounds against the malarial active site, lysyl-tRNA synthetase (PfKRS1).

## Material and methods

### Sponge collection

The Red Sea sponge *Callyspongia siphonella* (1.5 kg) was obtained in March 2021 at a depth of 7 m off the coast of Hurghada on the Red Sea (2,715,048″ north (N), 334,903″ east (E)). The Invertebrates Department, National Institute of Oceanography and Fisheries, Red Sea Branch, Hurghada, Egypt, was given a voucher sample (NIOF730/2021). The sponge was identified by El-Sayd Abed El-Aziz (Department of Invertebrates Lab., National Institute of Oceanography and Fisheries, Red Sea Branch, 84511 Hurghada, Egypt). Sponge biomass was transferred to plastic bag containing seawater and transported to the laboratory. Sponge specimens were rinsed in sterile seawater, cut into pieces of ca. ˜1 cm^3^, and then thoroughly homogenized in a sterile mortar with 10 volumes of sterile seawater. The supernatant was diluted in ten-fold series (10^-1^, 10^-2^, 10^-3^) and subsequently plated out on agar plates.

### Actinomycetes isolation

Three different media [M1, ISP-2 medium and Oligotrophic medium (OLIGO)] were used for isolation of actinobacteria. All media were supplemented with 0.2 µm pore size filtered cycloheximide (100 µg/mL), nystatin (25 µg/mL) and nalidixic acid (25 µg/mL) to facilitate the isolation of slow-growing actinobacteria. Cycloheximide and nystatin inhibit fungal growth, while nalidixic acid inhibits many fast-growing Gram-negative bacteria [8]. All media contained Difco Bacto agar (18 g/L) and were prepared in 1 L artificial sea water (NaCl 234.7 g, MgCl_2_.6 H_2_O 106.4 g, Na_2_SO_4_ 39.2 g, CaCl_2_ 11.0 g, NaHCO_3_ 1.92 g, KCl 6.64 g, KBr 0.96 g, H_3_BO_3_ 0.26 g, SrCl_2_ 0,24 g, NaF 0.03 g and ddH_2_O to 10.0 L). The inoculated plates were incubated at 30 °C for 6–8 weeks. Distinct colony morphotypes were picked and re-streaked until visually free of contaminants. 30 isolates were picked up upon morphological differences. The isolates were maintained on plates for short-term storage, and long-term strain collections were set up in medium supplemented with 30% glycerol at −80 °C.

### Molecular identification and phylogenetic analysis

16S rRNA gene amplification cloning and sequencing by Real-Time PCR (qRT-PCR) were performed as described in Cheng et al., (2015) [10] using the universal primers 27F and 1492R (Goldstar, Eurogentec, Seraing, Belgium) [11]. Chimeric sequences were identified by using the Pintail program [12]. The genus-level affiliation of the sequence was validated using the Ribosomal Database Project Classifier. The genus-level identification of all the sequences was done with RDP Classifier (-g 16srrna, -f allrank) and validated with the SILVA Incremental Aligner (SINA) (search and classify option). An alignment was calculated again using the SINA web aligner (variability profile: bacteria) [13]. Gap-only position were removed with trimAL (-noallgaps). For phylogenetic tree construction, the best fitting model was estimated initially with Model Generator. RAxML (-f a -m GTRGAMMA –x 12345 –p 12345 -# 1000) and the estimated model was used with 1000 bootstrap resamples to generate the maximum-likelihood tree [14]. Visualization was done with TreeGraph3 [15].

### Phylogenetic analysis

Nucleotide sequences of 16S rRNA of type strains of *Streptomyces* species and the outgroup *Nocardioides sediminis* were extracted from EZTAXON database https://www.ezbiocloud.net/ . The sequences from *Streptomyces* type strains were chosen based on the highest percentage of similarity against the query sequence sequenced in this study (Strain US4). The 16S rRNA sequences were aligned using Muscle (10 maximum number of iterations) implemented in Geneious Prime 2022.1.1. https://www.geneious.com/. The alignment was manually curated, insertions were trimmed and the best nucleotide model determined using MEGAX [16]. Maximum Likelihood tree was computed using MEGA X with 1000 bootstrap replicates and using the best model (TN93+G+I = Tamura Nei + Gamma Distribution + Invariant sites). The accession number for the 16S rRNA gene sequence of strain US4 is: OQ739151.

### Extract preparation

The isolated strain was cultivated using three different production media (M1, ISP2, OLIGO), static and shaking approaches to produce 6 organic extracts. The liquid cultures were grown for 10 days at 30 °C while shaking at 150 rpm. The culture was then filtered, and supernatant was extracted with ethyl acetate, while the cells and mycelia were extracted by shaking with methanol for 4 hours. The ethyl acetate extracts were stored at 4 °C.

### LC-MS profiling

Samples were prepared and analyzed as previously described by Gamaleldin *et al*. [8]. For mass spectrometry analysis, three extracts from the samples were produced using ethyl acetate and three using methanol, both at a concentration of 1 mg/mL. According to Abdelmohsen et al. 2014 [17], the recovered ethyl acetate extracts were subjected to metabolic investigation using LC-HR-ESI-MS. We employed a Synapt G2 HDMS quadrupole time-of-flight hybrid mass spectrometer (Waters, Milford, USA) coupled to an Acquity Ultra Performance Liquid Chromatography system. On an Accela HPLC (Thermo FisherFisher Scientific, Bremen, Germany), extracts diluted to 1 mg/mL were examined in triplicates. The HPLC column was an ACE (Hichrom Limited, Reading, UK) C18, 75 mm x 3.0 mm, 5 µm column. The mobile phase composed of purified water (A) and acetonitrile (B), each containing 0.1% formic acid. The gradient programme began with 10% B, raised B linearly in 30 min to 100% B at a flow rate of 300 µL/min, remained isocratic for 5 min, and then linearly decreased B to 10% B in 1 min. Before the subsequent injection, the column was re-equilibrated with 10% B for 9 min. Each sample's analysis took 45 minutes in total. The injection volume was 10 µL, and 12 °C was kept in the tray temperature [18]. The high-resolution mass spectrometry was performed using both positive and negative ESI ionization modes in conjunction with a spray voltage of 4.5 kV, a capillary temperature of 320 °C, and a mass range of m/z 150–1500. Based on the predetermined parameters, the MS dataset was analyzed, and data were retrieved using Mzmine 2.20. Chromatogram builder and chromatogram deconvolution were detected alongside mass ion peak detection. The isotopic peaks of grouper were used to differentiate isotopes, and the local minimum search algorithm was addressed. Using the gap-filling peak finder, missing peaks were highlighted. A complicated search and an adduct search were also conducted. Afterwards, peak identification and the prediction of the chemical formula were applied to the processed data set. The corresponding extract's positive and negative ionization mode data sets were dereplicated and compared to the DNP (Dictionary of Natural Products) databases. ChemBioDraw Ultra 14.0 software was used to create chemical structure drawings.

### Multivariate and statistical analysis

Data was prepared as illustrated by El-Hawary et al. [19] and uploaded to MetaboAnalyst 5.0 server (https://www.metaboanalyst.ca).

### Antimalarial assay

This assay was performed as previously described by Gamaleldin *et al*. [8]. The Malstat assay, which was described in [12, 13], was used to determine the extract's anti-plasmodial activity on *P. falciparum* erythrocytic replication in vitro. *P. falciparum* NF54 strains were used to plate synchronized ring-stage parasites with 1% parasitaemia in triplicate in 96-well plates (200 L/well) in the presence of successive dilutions of extracts diluted in 0.5% v/v dimethyl sulfoxide (DMSO). The extracts were cultured with the parasites for 72 hours at 37 °C with (90 % N_2_, 5 % O_2_, and 5 % CO_2_. The parasites were incubated with DMSO at a concentration of 0.5 % alone as a negative control, and the parasites were incubated with 20% DMSO as a positive control. Afterwards, 20 L was taken out and put on a fresh 96-well microtiter plate with 100 L of the Malstat reagent (1% Triton X-100, 10 mg of l-lactate, 3.3 mg Tris, and 0.33 mg of 3-Acetylpyridine adenine dinucleotide dissolved in 1 mL of distilled water, pH 9.0). A 20 L mixture of NBT (Nitro Blue Tetrazolium)/Diaphorase (1:1; 1 mg/mL stock each) was then added to the Malstat reaction to measure the plasmodial lactate dehydrogenase activity. Using the fifth version of the GraphPad Prism programme, the optical densities were determined at 630 nm, and the IC_50_ values were computed from variable-slope sigmoidal dose-response curves.

### Ligand design and docking

In this study, based on this fact that the ligands structures were not exist in PubChem. Avogadro 1.2.0 [20] was used to design the ligands. Molecular dockings of the selected small molecules were carried out by AutoDock 4.2 [21] in order to investigate the binding affinity of the ligands to the protein. AutoDockTools (ADT) 1.5.6 was utilized to prepare the docking inputs files. All hydrogens were added, and Kolman charges were calculated. Afterward, each non-polar hydrogen was merged with its corresponding carbon atom. Followed by specifying the torsion tree, small molecules were saved in PDBQT format and utilized for local docking. The grid box was adjusted to 60 × 50 × 70 Å points in xyz directions with 375 Å spacing set on the ligand-binding site. The LGA was applied using the default values except for the number of GA runs, which considered 500. Docking was performed on a rigid receptor, and small molecules were regarded as flexible. Later, the binding modes of the complex structures were evaluated by Chimera software [22]. Eventually, the best binding modes were selected.

## Results

### Phylogenetic analysis of the strain *Streptomyces* sp. US4

**Fig. 1:**
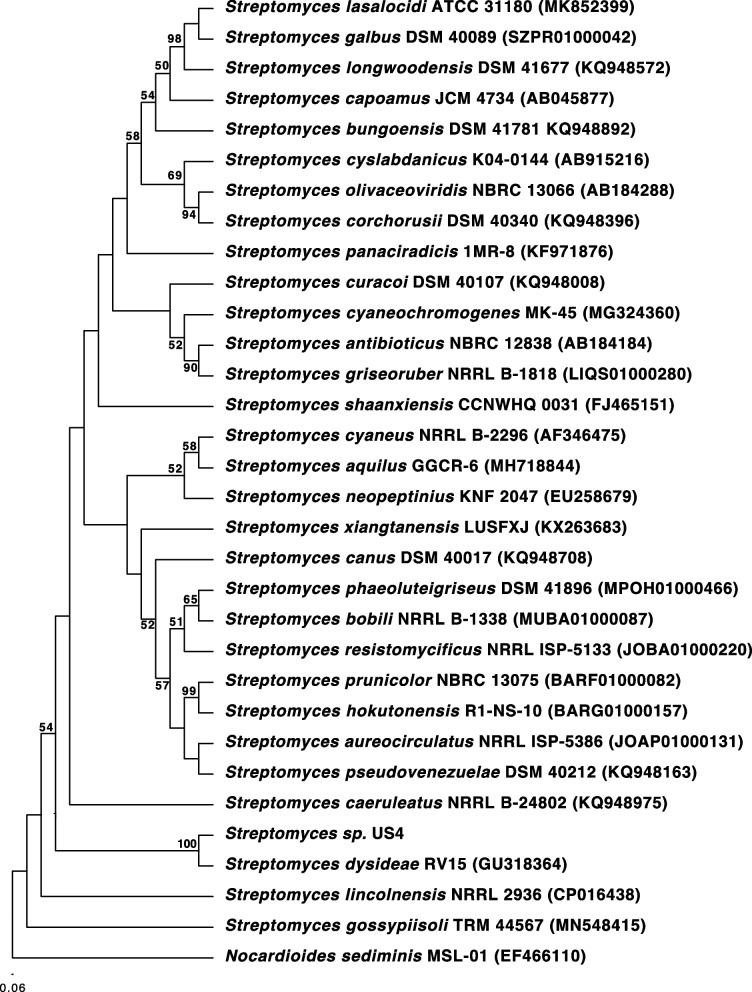
Neighbour-joining phylogenetic tree based on 16S rRNA sequences of the actinomycete isolate *Streptomyces* US4 and related sequences. The accession number of strain *Streptomyces* sp. US4 is OQ739151

Taxonomic affiliation of the isolated strain (US4) was conducted by comparing 16S rRNA sequences from *Streptomyces* closest type strains. The sequenced strain US4 has the highest 16S rRNA similarity with the type strain *Streptomyces dysideae* sharing 96% similarity. A phylogenetic tree including the closest 30 type strains from *Streptomyces* described species verified the supported clade of these two strains (Fig. 1) with a 100% bootstrap support. The cut-off of 98.63% for 16S rRNA species delineation [23] suggest that the strain US4 could represent a new species within this genus. Further analysis will need to be conducted to describe this strain as a novel species.

### Antimalarial assay

The culture extracts were tested *in vitro* for their antimalarial activities against the pathogenic, *Plasmodium falciparum* strain and results were presented in (Table 1)*.* Results revealed that the extracts, ISP2-S and ISP2-L exhibited the highest inhibitory activity with IC_50_ values 2.7 and 9.8 µg/mL, respectively. While, M1-S and M1-L showed moderate anti-plasmodial activity with IC_50_ values of 11.5 and 18.4 µg/mL, respectively. However, Oligo-S and Oligo-L were inactive compared to the positive control drug chloroquine (IC_50_ value: 0.022 µg/mL).

**Table 1:** In-vitro antimalarial activity of *Streptomyces* strain fermented extracts against *Plasmodium falciparum*

Code	Sample	IC_50_ (µg/ml)
1	**M1-S**	11.5
2	**ISP2-S**	2.7
3	**Oligo-S**	> 50
4	**M1-L**	18.4
5	**ISP2-L**	9.8
6	**Oligo-L**	> 50
7	**Chloroquine**	0.022

### Metabolomics and multivariate data analysis

#### Unsupervised analysis

**Fig. 2:**
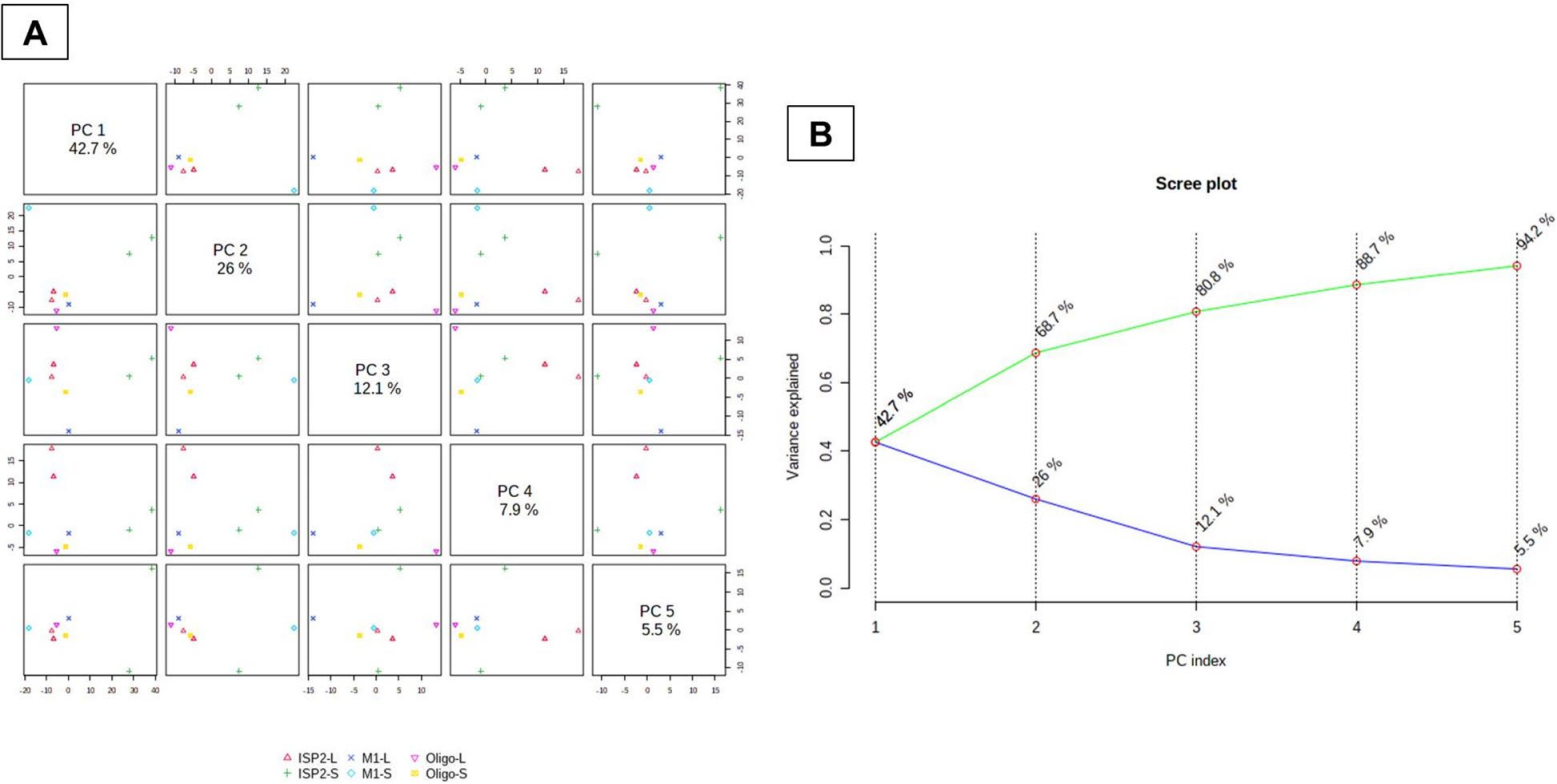
**A** PCA pairwise score plot of the unsupervised method; (**B**) PCA scree plot of the unsupervised method

**Fig. 3:**
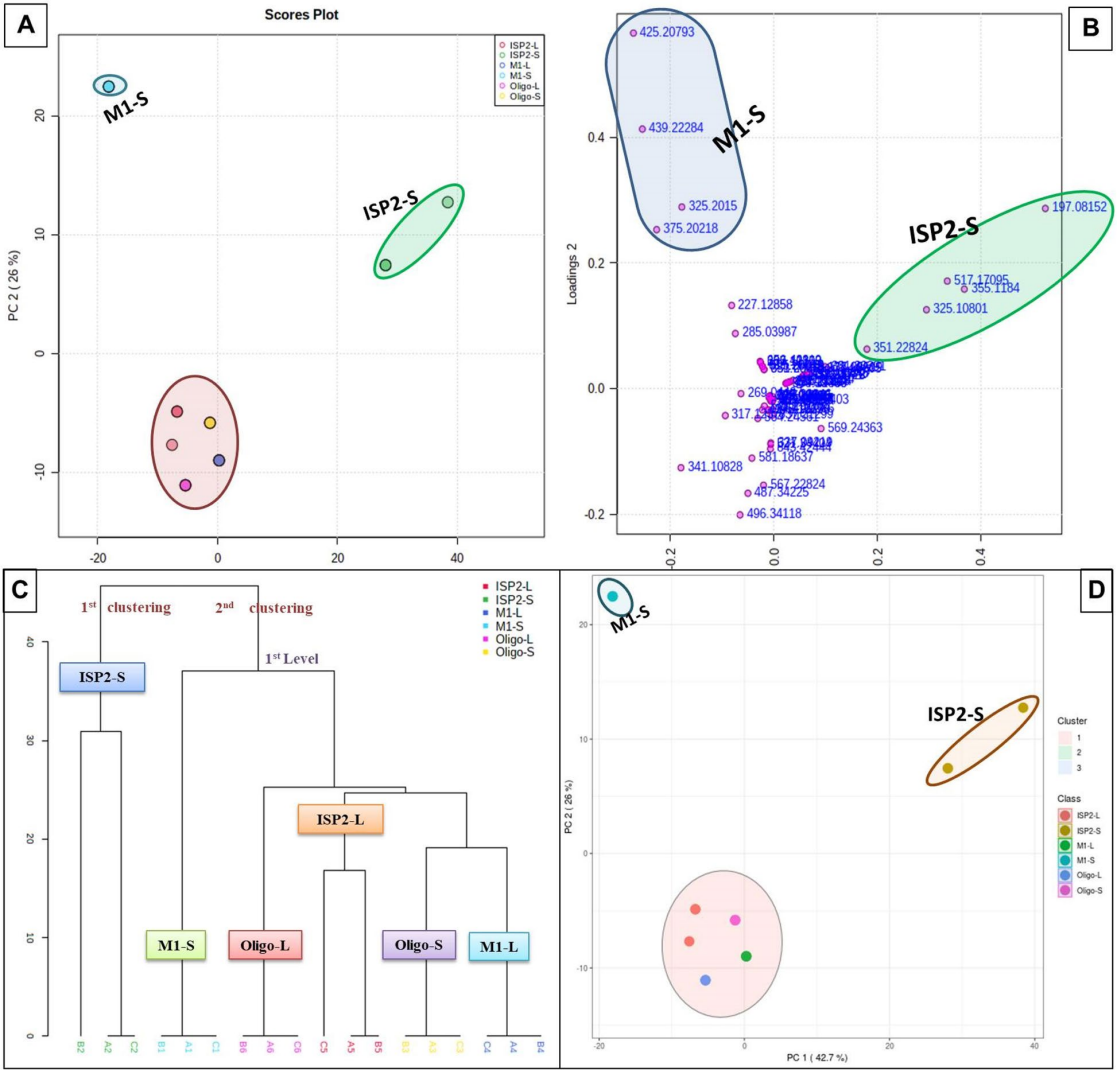
Metabolomics multivariate analysis. **A** 2D PCA scores plot of the unsupervised method; (**B**) 2D PCA loadings plot of the unsupervised method; (**C**) HCA plot showed as dendogram; (**D**) K-means clustering analysis

The PCA pairwise score graphs and scree plot (Fig. 2) revealed that there are five PCA components (PCs) explained 94.2% of the total variation, in which the first and second PCs individually contributed to 68.7% of the total variation (PC1 and PC2 represent 42.7% and 26%, respectively (Fig. 2B). In PCA 2D scores plot (Fig. 3A), the samples were distributed to three separated areas between PC1 and PC2, which indicated statistically significant differences between the extracts; the outliers were for the samples ISP2-S and M1-S (Fig. 3A). The PCA loadings plot (Fig. 3B) indicated the metabolites (m/z) that contributed to the variation of the anomalous samples. Dictionary of Natural Products (DNP) was used to annotate such metabolites; the annotated discriminatory compounds for ISP2-S corresponding to m/z (retention time in min.) 197.08152 [M^_^H]^_^ (2.701041) was identified as; 2-Butyl-4-(hydroxymethyl)-3-furancarboxylic acid (C_10_H_14_O_4_) [24] or; 4-(Hydroxymethyl)-2-(2-methylpropyl)-3-furancarboxylic acid (C_10_H_14_O_4_) [25]. The mass ion peak at m/z 355.1184 [M^_^H]^_^ (RT, 3.9897 min), corresponding to the proposed molecular formula C_20_H_20_O_6_, was identified as antibiotic X 14881B [26]. The mass ion peak at m/z 325.10801 [M^_^H]^_^ (RT, 4.0799 min), in accordance with the molecular formula C_19_H_18_O_5_ was recognized as 1,3,6-trihydroxy-8-(3-methylbutyl)anthraquinone [27]. Whereas that at m/z 517.1709 [M ^_^ H] ^+^ (RT, 4.4354 min) corresponding to the molecular formula C_26_H_28_O_11_ was suggested to be didemethylmutactimycin [28] or 8-demethoxy-2'-de-O-methylsteffimycin D [29], On the other hand, the annotated discriminatory compounds for M1-S are: The mass ion peak corresponding to m/z (retention time in min.) 425.20793 [M^_^H] ^+^ (3.6336) was putatively identified as JBIR 107 [30] or streptophenazine B or streptophenazine A [31]. (C_24_H_28_N_2_O_5_). The mass ion peak at m/z 439.22284 [M+H] ^+^ (RT, 4.2521 min), corresponding to the proposed molecular formula (C_25_H_30_N_2_O_5_), was identified as streptophenazine F or streptophenazine G [31]. Where that at m/z 375.2021 [M ^_^ H] ^+^ (RT, 3.8968 min) corresponding to the molecular formula (C_18_H_30_O_8_) was suggested to be 2-hydroxy, 2,3-dihydro-antibiotic SEN 366D1. Finally, the mass ion peak at 325.2015 [M^_^H]^_^ (RT, 3.8514) corresponding to the molecular formula (C_18_H_30_O_5_) was annotated as Albocycline M6 or Albocycline M3 or Antibiotic A121 [32, 33].

#### Clustering analysis

The hierarchical clusters analysis (Fig. 3C) and the K-means clustering analysis (Fig. 3D) also revealed the diversity of the samples, ISP2-S and M1-S. The diagrams showed a unique variation of the samples, ISP2-S and M1-S, which indicating their chemical diversity from other samples and from each other (Fig. 3).

#### Supervised analysis

### Partial Least Squares - Discriminant Analysis (PLS-DA)

**Fig. 4:**
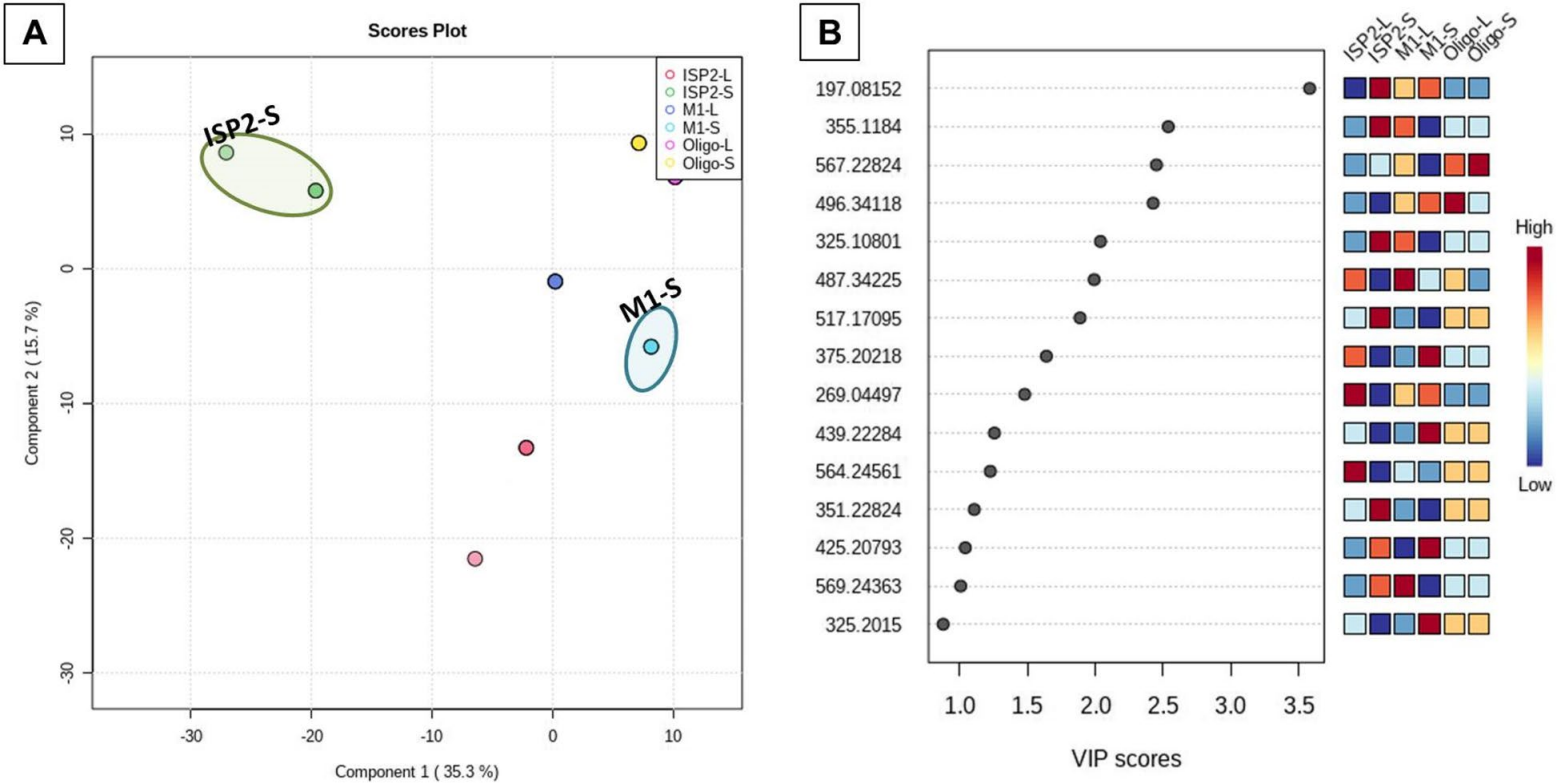
Metabolomics multivariate analysis. **A** PLS-DA scores plot; (**B**) VIP score plot of PLS-DA

**Fig. 5:**
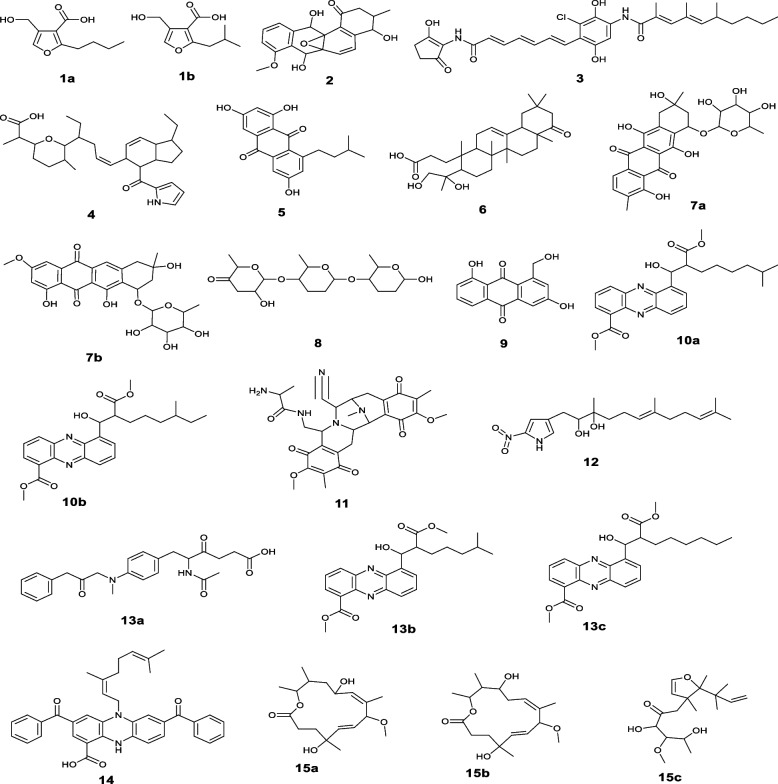
Chemical structures of the 15 most important VIPs

The created model performed well in terms of both prediction (predictive power of models, Q2 = 0.9) and performance (model goodness, R2 = 0.99), a high Q2 value is indicative of good predictions (Q2 values were those that were very near to 1.0). The score plot of partial least squares discriminant analysis (PLS‐DA) revealed a noticeable separation between the sample groups indicating a significant change in the metabolite profile (Fig. 4A); the samples ISP2-S and M1-S were outliers. One of the crucial measurements in PLS-DA is the variable Importance in Projection (VIP), which can be seen in Fig. 4B and Table 2. VIP demonstrated the most 15 important features of highest value identified by PLS-DA (Table 2) (Fig. 5).

**Table 2:** The 15 most important VIPs

No	Ionization	m/z	Rt	Name	formula	Source	Class	Reference
1	N	197.0815	2.70104	**(a)**2-Butyl-4-(hydroxymethyl)-3-furancarboxylic acid	C_10_H_14_O_4_	*Streptomyces coelicolor*	Lactones	[24]
				**(b)**4-(Hydroxymethyl)-2-(2-methylpropyl)-3-furancarboxylic acid				[25]
2	N	355.1184	3.98977	Antibiotic X 14881B	C_20_H_20_O_6_	*Streptomyces sp.*	Anthracene	[27]
3	N	567.2282	3.652877	Chinikomycin A	C_31_H_37_C_l_N_2_O_6_	*Streptomyces lavendulae*	Manumycins	[49]
4	p	496.3411	5.947817	8,9-Dihydroindanomycin	C_31_H_45_NO_4_	*Streptomyces galbus*	Quinones	[50]
5	N	325.1080	4.079911	1,3,6-Trihydroxy-8-(3-methylbutyl) anthraquinone	C_19_H_18_O_5_	*Streptomyces No. 1128*		[51]
6	N	487.3422	4.804455	4,23-Dihydroxy-22-oxo-3,4-seco-12-oleanen-3-oic acid	C_30_H_48_O_5_	*Streptomyces sp. GT 44003*	Triterpenoids	[52]
7	P	517.1709	4.435446	**(a)**Didemethylmutactimycin	C_26_H_28_O_11_	*Streptomyces sp. GW 60/157*	Quinones	[28]
				**(b)**8-Demethoxy-2'-de-O-methylsteffimycin D		*Streptomyces steffisburgensis NRRL 3193*		[29]
8	P	375.2021	3.8968476	Antibiotic SEN 366D1; 2-Hydroxy, 2,3-dihydro	C_18_H_30_O_8_	*Streptomyces sp. SEN366-BP577*		
9	N	269.0449	3.701269	ω-Hydroxyaloesaponarin II	C_15_H_10_O_5_	*Streptomyces sp. M097*	Quinones	[53]
10	p	439.2228	4.252196	**(a)**Streptophenazine F	C_25_H_30_N_2_O_5_	*marine-derived Streptomyces sp. strain HB202*	Phenazines	[31]
				**(b)**Streptophenazine G				
11	P	564.2456	3.511726	Saframycin Y3	C_29_H_33_N_5_O_7_	*Streptomyces lavendulae*	Quinones	[47]
12	P	351.2282	4.029025	Nitropyrrolin A	C_19_H_30_N_2_O_4_	*marine-derived Streptomyces sp. CNQ-509*	Nitropyrrolins	[54]
13	P	425.2079	3.6336655	**(a)**JBIR 107	C_24_H_28_N_2_O_5_	*marine-derived Streptomyces tateyamensis NBRC 105047*		[30]
				**(b)**Streptophenazine A		*marine-derived Streptomyces sp. strain HB202*	Phenazines	[31]
				**(c)**Streptophenazine B				
14	N	569.2436	3.472232	Benthophoenin	C_37_H_34_N_2_O_4_	*Streptomyces prunicolor*	Phenazines	[55]
15	N	325.2015	3.8514833	**(a)**Albocycline M6	C_18_H_30_O_5_	*Streptomyces bruneogriseus*	Macrolides	[32]
				**(b)**Albocycline M3				
				**(c)**Antibiotic A 121		*Streptomyces sp.*	non-polyene antibiotic	[33]

### The Orthogonal Projections to Latent Structures Discriminant Analysis (OPLS-DA)

**Fig. 6:**
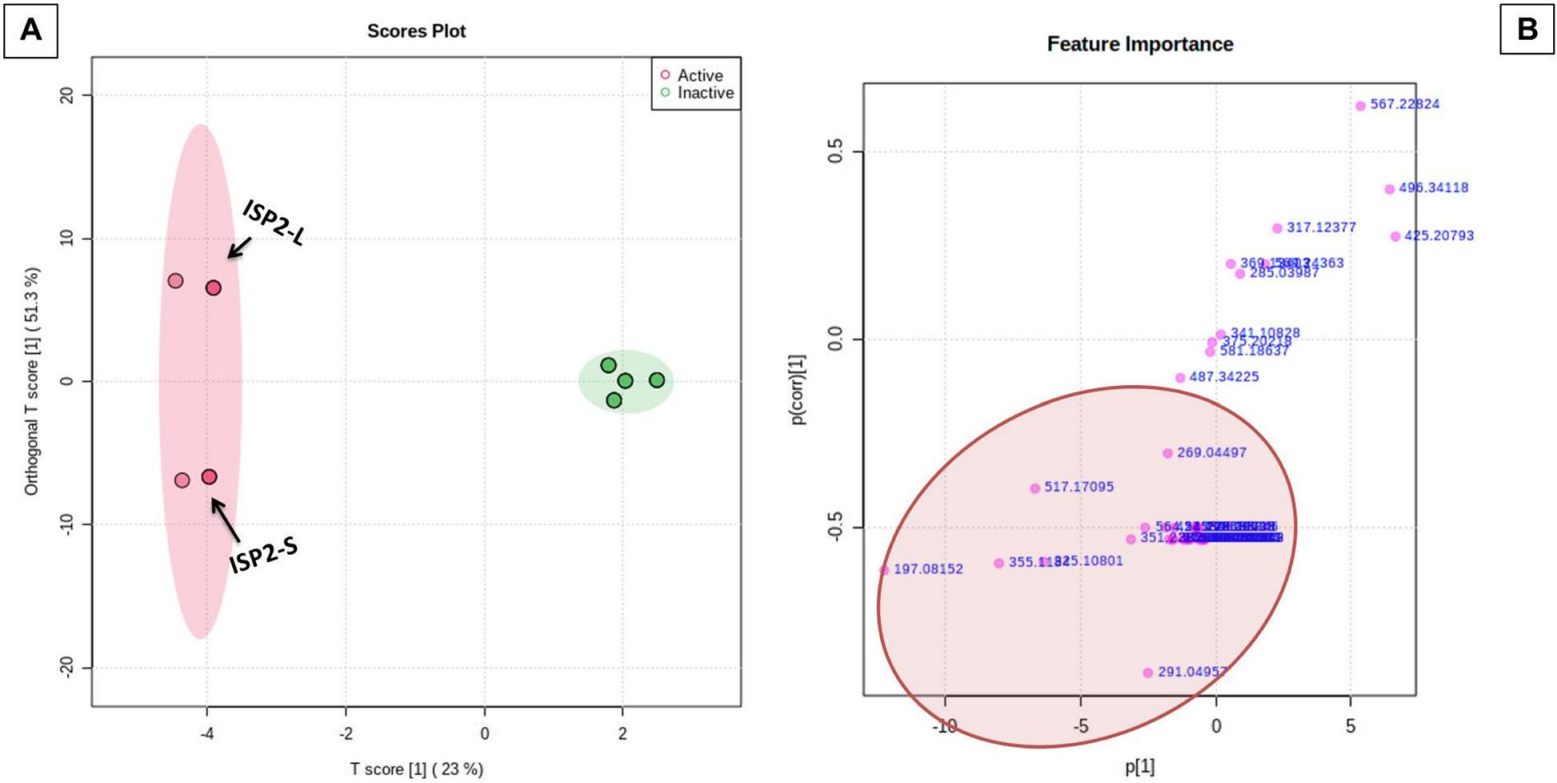
Metabolomics multivariate analysis; (**A**) OPLS-DA score plot; (**B**) OPLS-DA S-plot demonstrating the masses of the most important metabolites (1–8) that may be associated with the anti-plasmodial activity

OPLS-DA method was carried out to investigate the link between *Streptomyce*s derived extract's anti-plasmodial efficacy and their metabolite profiles. The developed model performed well in terms of performance (R2 = 0.99) and prediction (Q2 = 0.989) (*P* < 0.5); the best R2 values were those that were extremely near to 1.0 [34]. The most active extracts, with IC_50_ values less than 10 µg/ml (ISP2-S and ISP2-L) were classified as active anti-plasmodial. Whereas those with higher values (>10 µg/ml) were classified as inactive. The results were presented in OPLS-DA score plot (Fig. 6) (Table 3).

**Table 3:** Metabolites that correlated to the observed antimalarial activity of the active extracts as predicted using the OPLS-DA-derived S-Plots

	**Mode**	**m/z**	**Rt**	**Name**	**formula**	**Key fragments**	**Source**	**Class**	**Ref**
**1**	P	291.049	2.117525	Juglomycin E	C_14_H_10_O_7_	273.18; 171.10	*Streptomyces* sp.	Quinones	[44]
**2**	P	517.170	4.4354464	Didemethylmutactimycin	C_26_H_28_O_11_	417	*Streptomyces sp. GW 60/157*	Quinones	[28]
**3**	N	325.108	4.0799119	1,3,6-Trihydroxy-8-(3-methylbutyl) anthraquinone	C_19_H_18_O_5_	323.39; 297.7; 281.11	*Streptomyces No. 1128*	Quinones	[51]
**4**	N	269.044	3.7012692	ω-Hydroxyaloesaponarin II	C_15_H_10_O_5_	225.10; 177.05; 213.11	*Streptomyces sp. M097*	Quinones	[53]
**5**	P	564.245	3.5117262	Saframycin Y3	C_29_H_33_N_5_O_7_	502.17; 461.10; 415.19	*S. lavendulae*	Quinones	[47]
**6**	N	355.118	3.9897705	X 14881B	C_20_H_20_O_6_	–	*Streptomyces sp. Y-83,30,683*	Anthracene	[27]
**7**	N	197.081	2.701041	2-Butyl-4-(hydroxymethyl)-3-furancarboxylic acid	C_10_H_14_O_4_	133.54; 91.41; 57.28	*S. coelicolor*	Lactones	[24]
**8**	P	351.228	4.029025	Nitropyrrolin A	C_19_H_30_N_2_O_4_	249.18; 197.12; 67.05	*Streptomyces sp. CNQ-509*	Nitropyrrolins	[54]

#### Molecular docking study

**Fig. 7:**
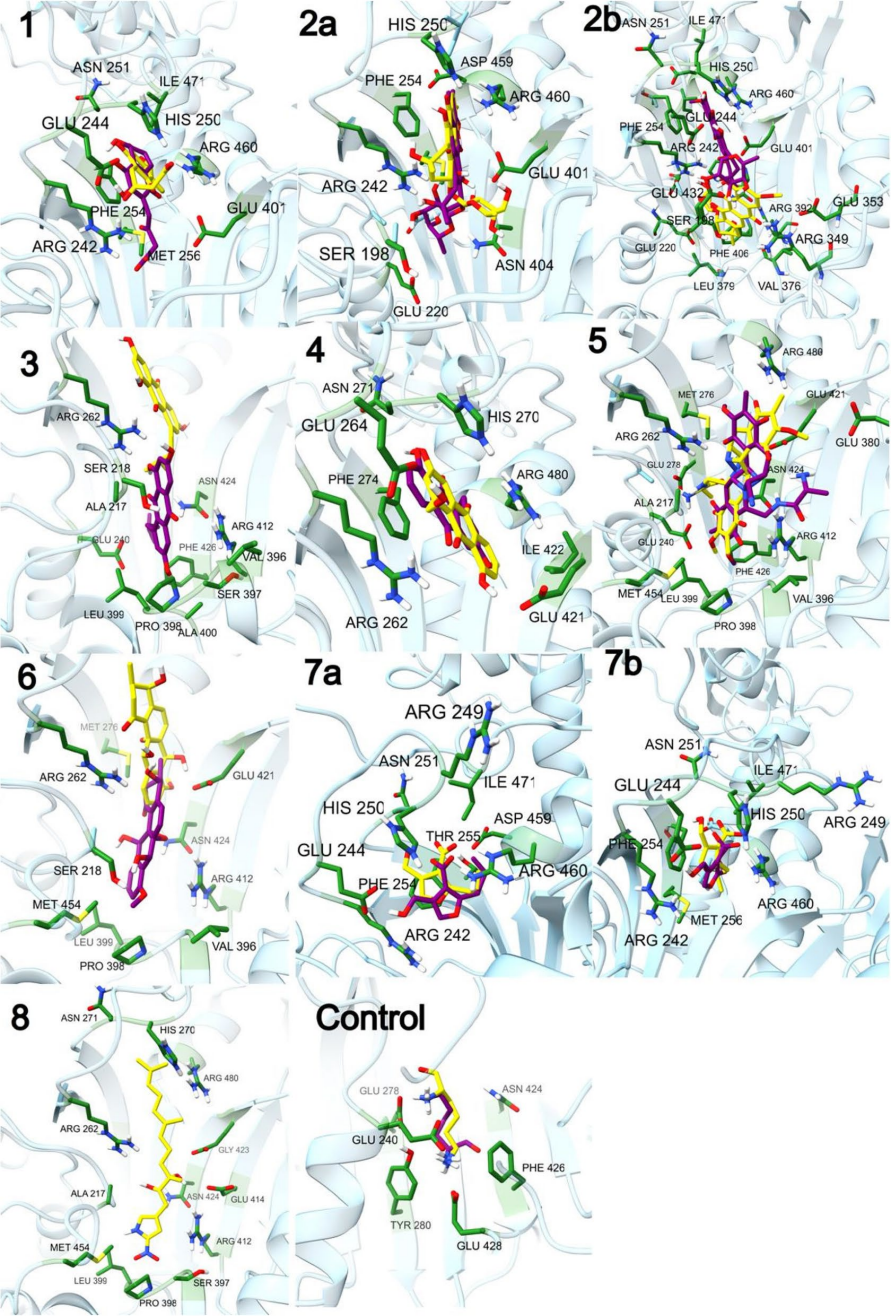
3D structures of interaction between lysyl-tRNA synthetase and ten selected ligands

Molecular docking study was employed in order to assess the binding modes and calculate the binding affinity between lysyl-tRNA synthetase (PfKRS1) and the selected small molecules. Initially, this protein (PDB code: 1lyl), was docked into the binding site of ten selected ligands respectively including, juglomycin E (1), didemethylmutactimycin (2a), 8-demethoxy-2'-de-O-methylsteffimycin D (2b), 1,3,6-trihydroxy-8-(3-methylbutyl) anthraquinone (3), ω-hydroxyaloesaponarin II (4), saframycin Y3 (5), antibiotic X 14881B (6), 2-butyl-4-(hydroxymethyl)-3-furancarboxylic acid (7a), 4-(hydroxymethyl)-2-(2-methylpropyl)-3-furancarboxylic acid (7b) and nitropyrrolin A (8). The binding energy scores and intermolecular interaction information of 1lyl and selected ligands are tabulated in Table 4. 3D structures of interaction between lysyl-tRNA synthetase and ten selected ligands are shown in Fig. 7.

**Table 4:** Docking results for all ligands

	NO. of structures	Binding Energy	Ligand Efficiency	Ki (***μ***M)	Intermol Energy	VdW Energy	Elec. Energy
Control	64	-7.34	-0.82	4.17	-9.43	-4.09	-5.34
1	51	-5.13	-0.24	175.07	-6.91	-5.39	-1.53
2a	282	-4.01	-0.11	1150	-6.69	-5.71	-0.98
2b	102	-3.37	-0.09	3400	-6.05	-5.35	-0.7
3	92	-4.04	-0.17	1090	-5.83	-5.58	-0.25
4	180	-4.82	-0.24	295.42	-6.01	-5.34	-0.67
5	95	-6.2	-0.16	28.66	-7.99	-3.89	-4.1
6	58	-3.23	-0.12	4300	-4.42	-4.26	-0.16
7a	142	-3.93	-0.28	1310	-5.72	-4.78	-0.95
7b	130	-3.89	-0.28	1410	-5.38	-4.39	-0.99
8	500	-0.83	-0.03	246,720	-4.41	-4.25	-0.16

All metabolites demonstrated binding affinity to lysyl-tRNA synthetase binding pocket, with energy values ranging from strong (-6.2) to weak (-3.23). Ten compounds were evaluated, and the quinone derivatives saframycin Y3 and juglomycin E exhibited the highest values (-6.2and -5.13, respectively) followed by ω-hydroxyaloesaponarin II with energy score -4.82. While, antibiotic X 14881B ranked the lowest energy score (-3.23) as presented in Table 4.

## Discussion

The scientific community is always fascinated by natural products. Natural products now contain distinctive chemical heterogeneity as a result of evolution over millions of years, giving them drug-like qualities and a variety of biological activities. They have offered a number of medicinal lead compounds. A significant number of these lead chemicals were identified from microbial populations. Actinobacteria are a substantial source of medically relevant chemicals, including anti-infective medicines; It contributed over 80% of the antibiotics currently used in medicine, with *Streptomyces* accounting for 50% of those compounds [35]. As a result of our continuous attempts to find bioactive natural compounds with antimalarial potential, the actinomycete associated with the Red Sea sponge *Callyspongia siphonella* was isolated and identified as *Streptomyces* sp. US4 . Using OSMAC approach, three different media (M1, ISP2 and OLIGO) were used to subculture the isolated strain in order to activate silent and poorly expressed gene clusters. Extracts were screened in-vitro against *Plasmodium falciparum* strain, and since the antimalarial results were effective, in particular for the extract ISP2-S, this encouraged us to explore their chemical constituents using the most popular efficient method, untargeted LC-HR-MS metabolomic profiling. The obtained LC-HR-MS data was statistically treated using MetaboAnalyst- a Multivariate data statistical analysis (MVA) platform- in order to reduce the vast amount of data obtained and detect correlation and differentiation among the tested samples [36, 37]. The unsupervised principal component analysis (PCA) method and the supervised method Partial Least Squares- Discriminant Analysis (PLS-DA) revealed that the culture extracts ISP2-S and M1-S were dispersed from the others indicating there chemical diversity, which is further corroborated by their distinct patterns in the heatmap plot.

**Fig. 8:**
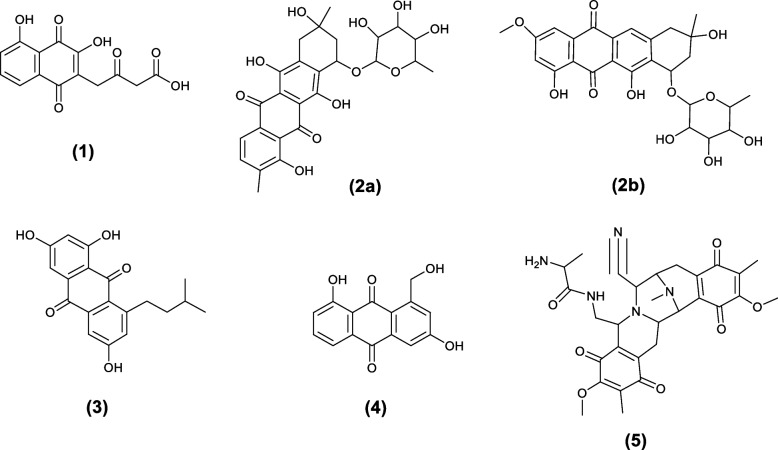
Structures of metabolites (1–5) that were highly correlated to the extracts’ anti-malarial activity

**Fig. 9:**
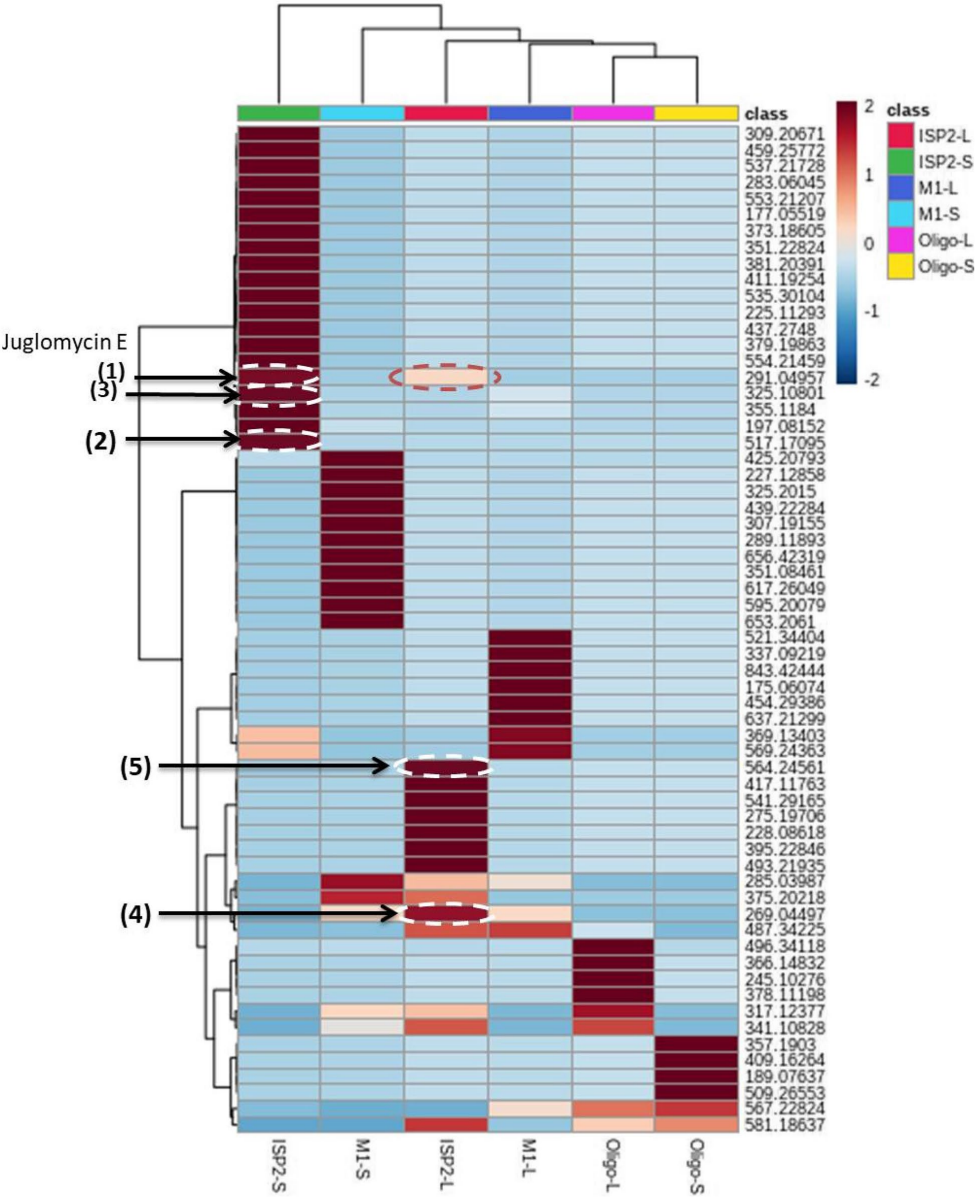
Heat-map indicating the distribution and abundance of quinones (1–5) in the active antimalarial extracts

The relation between the extracts and their antimalarial activity was investigated using OPLS-DA method. As shown in the OPLS-DA score plot (Fig. 6A) there are clear differences between active and inactive extracts. Where, active extracts clustered together, indicating that they contain related metabolites that could be responsible for their remarkable antiplasmodial activity. The bioactive discriminatory metabolites that may be related to the observed antimalarial activity of the active extracts were predicted using the OPLS-DA-derived S-Plots (Fig. 6B) (Table 3). Eight metabolites were dereplicated using the Dictionary of Natural Products (DNP), with Quinone derivatives (1–5) being the predominant metabolites (Table 3) (Fig. 8). The distribution of these bioactivity-linked metabolites among the extracts studied was depicted by the heatmap in Fig. 9; it was revealed that Juglomycin E (1) (C_14_H_10_O_7_) corresponding to m/z (retention time in min.) 291.04957 [M^_^H] ^+^ (2.1175) is common in the most active extracts, ISP2-S and ISP2-L.

The aforementioned findings revealed the abundance of quinone derivatives in the most active extracts. Since various studies revealed that drugs containing quinone nuclei exhibited promising anti-malarial activities [1, 28], it was crucial to apply docking analysis to examine these chemical compounds in more detail, determine their mode of action, and look into the connection between the chemical components and the resulting antimalarial activity. On the other hand, the focus on target-based drug discovery and finding targeted antimalarial drugs that treat all stages of the disease became a need. Previously published data revealed the presence of various malarial targets that affects different stages of parasite infection [8], among them, lysyl-tRNA synthetase, [38] which is an enzyme central to protein translation and responsible for protein formation [39]. Earlier studies revealed that lysyl-tRNA synthetase could be considered as an attractive, druggable, antimalarial target that can be selectively inhibits protein synthesis and prevents liver and blood-stage proliferation [34–38]. Therefore, computational prediction – Docking analysis – was performed in order to screen the annotated quinone derivatives against the antimalarial target, lysyl-tRNA synthetase.

Docking analysis is considered an alternative approach to drug discovery and is preferable to conventional drug discovery efforts that is time consuming, and is often implemented first to screen compounds with promising activity against different protein targets and then to identify their specific targets in order to ascertain their mode of action [39, 40]. Interestingly, all selected metabolites showed binding affinities ranging from (-6.2 and -3.23). Saframycin Y3 (5) and juglomycin E (1), two quinone derivatives, showed the highest values (-6.2 and -5.13, respectively), followed by ω-hydroxyaloesaponarin II (4), which had an energy score of -4.82. Therefore, among the variety of chemical components present in the analyzed extracts, the *in silico* docking method prioritized the putative active metabolites Saframycin Y3 (5) and juglomycin E (1) as promising antimalarial candidates.

Juglomycin E (1) is a derivative of juglomycins [41], which are broad spectrum naphthoquinone antibiotics naturally produced by different *Streptomyces* species [42, 43]. Saframycin Y3 (5) is a naturally produced active compound belongs to saframycins, which are heterocyclic quinone groups [44]. Saframycins are well known antitumor antibiotics produced by the actinomycetes strain *Streptomyces lavendulae* [45]*.*

## Conclusion

The marine bacterium, *Streptomyces* sp. was investigated utilizing the established process using LC-HR- MS-based analytical techniques. The OSMAC technique was used to isolate and subculture the actinomycete, *Streptomyces* associated with the Red Sea sponge *Callyspongia siphonella* in three distinct media (M1, ISP2, and OLIGO). The extracts were then examined for their antimalarial effectiveness and chemical diversity. The ISP2 extract showed significant antimalarial efficacy. On the other hand, many metabolites of diverse structural types, primarily quinones, have been found as a result of our study of this species. In silico studies revealed that the metabolites saframycin Y3 (5) and juglomycin E (1) have a high and comparable docking score within all other dereplicated molecules. The focus of this work was on the special role of actinomycetes related to marine species as an untapped source of active metabolites for the development of efficient natural antimalarial medicines. Finally, the journey of finding the best drug-like molecules is a difficult and continual process that takes a lot of time, however, this combination of tools and techniques acts as a model for the development of future targeted antimalarial drugs. Therefore, our research focuses on methods for accelerating the development of effective lysyl-tRNA synthetase inhibitors with therapeutic potential, and could be used to guide the creation of novel antimalarial drugs that can inhibit lysyl-tRNA synthetase. We suggest that future isolation and identification efforts to be only focus on the candidates that scored highest energy binding affinities in the docking study.

### Supplementary Information

**Additional file 1:** **Figure (S1).** LC/HRMS-negative mode total ion chromatogram of M1 solid extract (M1-S).  **Figure (S2).** LC/HRMS-positive mode total ion chromatogram of M1 solid extract (M1-S). **Figure (S3).** LC/HRMS-positive mode total ion chromatogram of ISP2 solid extract (ISP2-S). **Figure (S4).** LC/HRMS-negative mode total ion chromatogram of ISP2 solid extract (ISP2-S). **Figure (S5).** LC/HRMS-negative mode total ion chromatogram of Oligo solid extract (Oligo-S). **Figure (S6).** LC/HRMS-positive mode total ion chromatogram of Oligo solid extract (Oligo-S). **Figure (S7).** LC/HRMS-negative mode total ion chromatogram of M1 liquid extract (M1-L). **Figure (S8).** LC/HRMS-positive mode total ion chromatogram of the M1 liquid extract (M1-L). **Figure (S9).** LC/HRMS-negative mode total ion chromatogram of ISP2 liquid extract (ISP2-L). **Figure (S10).** LC/HRMS-positive mode total ion chromatogram of ISP2 liquid extract (ISP2-L). **Figure (S11).** LC/HRMS-positive mode total ion chromatogram of Oligo liquid extract (Oligo-L). **Figure (S12).** LC/HRMS-negative mode total ion chromatogram of Oligo liquid extract (Oligo-L).

## Data Availability

The whole genome sequences have been submitted to the NCBI/Nucleotide [accession number of strain *Streptomyces* sp. US4 is OQ739151]. Direct accessible link: https://www.ncbi.nlm.nih.gov/nuccore/OQ739151.1/.
