# Supplementary material for: Targeting antimalarial metabolites from the actinomycetes associated with the Red Sea sponge Callyspongia siphonella using a metabolomic method
Source: BMC Microbiol. 2023 Dec 12;23:396. doi: 10.1186/s12866-023-03094-3 (PMC10714608; doi:10.1186/s12866-023-03094-3)
Supplement: Supplementary file 1 — Additional file 1: Figure (S1). LC/HRMS-negative mode total ion chromatogram of M1 solid extract (M1-S). Figure (S2). LC/HRMS-positive mode total ion chromatogram of M1 solid extract (M1-S). Figure (S3). LC/HRMS-positive mode total ion chromatogram of ISP2 solid extract (ISP2-S). Figure (S4). LC/HRMS-negative mode total ion chromatogram of ISP2 solid extract (ISP2-S). Figure (S5). LC/HRMS-negative mode total ion chromatogram of Oligo solid extract (Oligo-S). Figure (S6). LC/HRMS-positive mode total ion chromatogram of Oligo solid extract (Oligo-S). Figure (S7). LC/HRMS-negative mode total ion chromatogram of M1 liquid extract (M1-L). Figure (S8). LC/HRMS-positive mode total ion chromatogram of the M1 liquid extract (M1-L). Figure (S9). LC/HRMS-negative mode total ion chromatogram of ISP2 liquid extract (ISP2-L). Figure (S10). LC/HRMS-positive mode total ion chromatogram of ISP2 liquid extract (ISP2-L). Figure (S11). LC/HRMS-positive mode total ion chromatogram of Oligo liquid extract (Oligo-L). Figure (S12). LC/HRMS-negative mode total ion chromatogram of Oligo liquid extract (Oligo-L). [file 12866_2023_3094_MOESM1_ESM.docx]

**Targeting antimalarial metabolites from the actinomycetes associated with the red sea sponge *Callyspongia siphonella* using a metabolomic method**

Noha M. Gamaleldin^1^, Hebatallah S. Bahr^2^, Natalie Millán-Aguiñaga^3^, Mahshid Danesh^4^, Eman M. Othman^4,5^, Thomas Dandekar^4^, Hossam M. Hassan^2^,^6*^, Usama Ramadan Abdelmohsen^7,8*^

^1^Department of Microbiology, Faculty of Pharmacy, the British University in Egypt (BUE), Cairo 11837, Egypt. noha.gamaleldin@bue.edu.eg

^2^Department of Pharmacognosy, Faculty of Pharmacy, Nahda University, Beni-Suef, Egypt.

^3^Universidad Autónoma de Baja California, Facultad de Ciencias Marinas. 22860 Ensenada, Baja California, México.

^4^Department of Bioinformatics, Biocenter, University of Würzburg, Am Hubland, 97074 Würzburg, Germany.

^5^ Department of Biochemistry, Faculty of Pharmacy, Minia University, 61519 Minia, Egypt.

^6^Department of Pharmacognosy, Faculty of Pharmacy, Beni-Suef University, Beni-Suef, Egypt.

^7^ Department of pharmacognosy, faculty of Pharmacy, Minia University, Minia, Egypt.

^8^ Department of pharmacognosy, faculty of Pharmacy, Deraya University, 61111 New Minia City, Minia, Egypt.

***Corresponding author:** [Hossam.mokhtar@nub.edu.eg](mailto:Hossam.mokhtar@nub.edu.eg) (HMH), [Usama.ramadan@mu.edu.eg](mailto:Usama.ramadan@mu.edu.eg) (URA)

**Abstract**

Malaria is a persistent illness that is still a public health issue. On the other hand, marine organisms are considered a rich source of anti‑infective drugs and other medically significant compounds. Herein, we reported the isolation of the actinomycete associated with the Red Sea sponge *Callyspongia siphonella*. Using "one strain many compounds" (OSMAC) approach, a suitable strain was identified and then sub-cultured in three different media (M1, ISP2 and OLIGO). The extracts were evaluated for their *in-vitro* antimalarial activity against *Plasmodium falciparum* strain and subsequently analyzed by Liquid chromatography coupled with high-resolution mass spectrometry (LC-HR-MS). In addition, MetaboAnalyst 5.0 was used to statistically analyze the LC-MS data. Finally, Molecular docking was carried out for the dereplicated metabolites against lysyl-tRNA synthetase (PfKRS1)*.* The phylogenetic study of the 16S rRNA sequence of the actinomycete isolate revealed its affiliation to *Streptomyces* genus. Antimalarial screening revealed that ISP2 media is the most active against *Plasmodium falciparum* strain. Based on LC-HR-MS based metabolomics and multivariate analyses, the static cultures of the media, ISP2 (ISP2-S) and M1 (M1-S)**,** are the optimal media for metabolites production. OPLS-DA suggested that quinone derivatives are abundant in the extracts with the highest antimalarial activity. Fifteen compounds were identified where eight of these metabolites were correlated to the observed antimalarial activity of the active extracts. According to molecular docking experiments, saframycin Y3 and juglomycin E showed the greatest binding energy scores (-6.2 and -5.13) to lysyl-tRNA synthetase (PfKRS1), respectively. Using metabolomics and molecular docking investigation, the quinones, saframycin Y3 **(5)** and juglomycin E **(1)** were identified as promising antimalarial therapeutic candidates. Our approach can be used as a first evaluation stage in natural product drug development, facilitating the separation of chosen metabolites, particularly biologically active ones.

**Keywords:** *Callyspongia siphonella*; *Streptomyces*; PCA; PLS-DA; antimalarial; metabolomics; quinones.

**Figure (S1):** LC/HRMS-negative mode total ion chromatogram of M1 solid extract (**M1-S).**

**Figure (S2):** LC/HRMS-positive mode total ion chromatogram of M1 solid extract (**M1-S).**

**Figure (S3):** LC/HRMS-positive mode total ion chromatogram of ISP2 solid extract (**ISP2-S**).

**Figure (S4):** LC/HRMS-negative mode total ion chromatogram of ISP2 solid extract (**ISP2-S**).

**Figure (S5):** LC/HRMS-negative mode total ion chromatogram of Oligo solid extract (**Oligo-S**).

**Figure (S6)**: LC/HRMS-positive mode total ion chromatogram of Oligo solid extract (**Oligo-S**).

**Figure (S7):** LC/HRMS-negative mode total ion chromatogram of M1 liquid extract (**M1-L).**

**Figure (S8):** LC/HRMS-positive mode total ion chromatogram of the M1 liquid extract (**M1-L).**

**Figure (S9):** LC/HRMS-negative mode total ion chromatogram of ISP2 liquid extract (**ISP2-L**).

**Figure (S10):** LC/HRMS-positive mode total ion chromatogram of ISP2 liquid extract (**ISP2-L**).

**Figure (S11):** LC/HRMS-positive mode total ion chromatogram of Oligo liquid extract (**Oligo-L**).

**Figure (S12):** LC/HRMS-negative mode total ion chromatogram of Oligo liquid extract (**Oligo-L**).


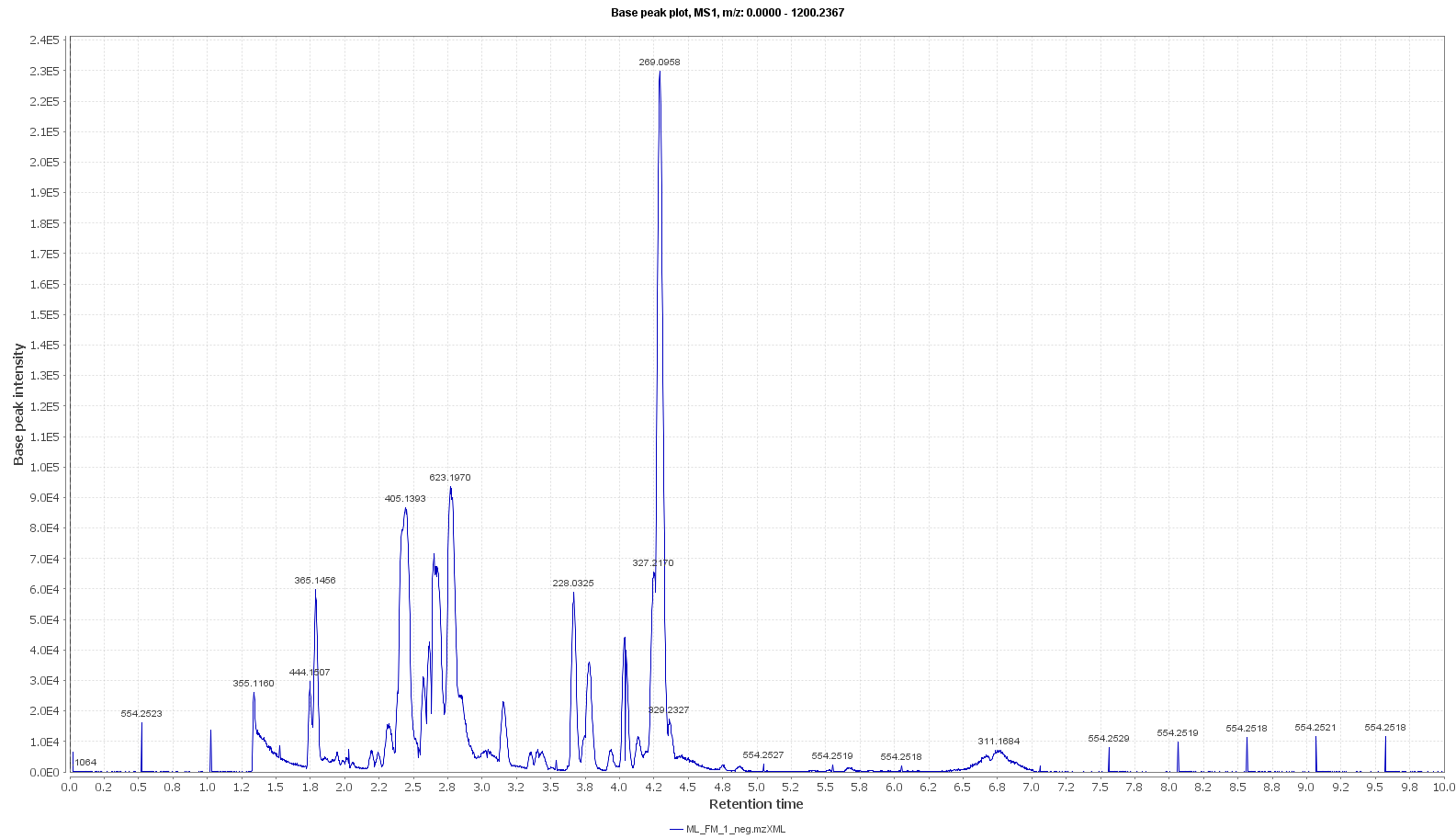
**Figure (S1):** LC/HRMS-negative mode total ion chromatogram of M1 solid extract (**M1-S).**


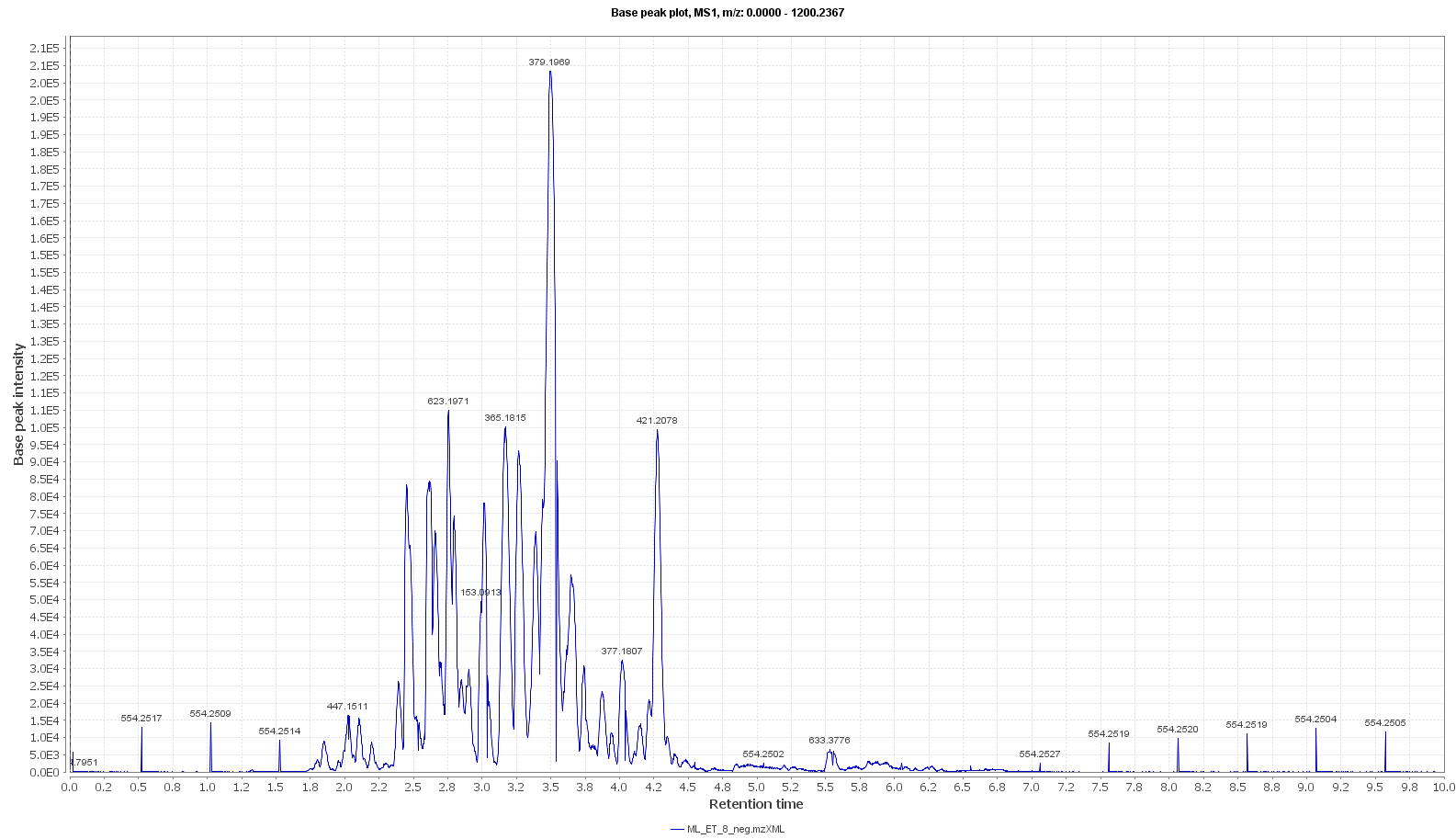
**Figure (S2):** LC/HRMS-positive mode total ion chromatogram of M1 solid extract (**M1-S).**


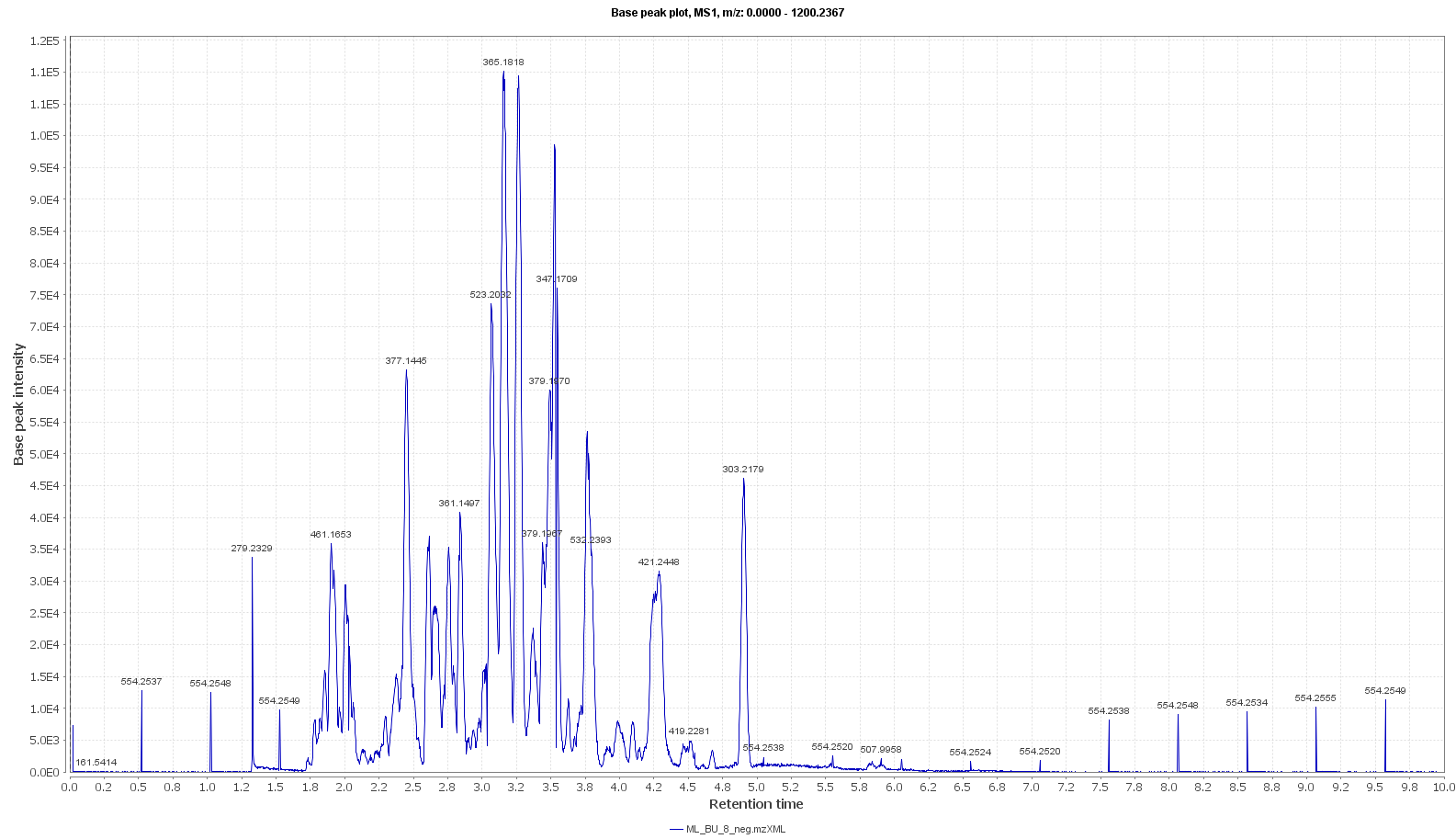
**Figure (S3):** LC/HRMS-positive mode total ion chromatogram of ISP2 solid extract (**ISP2-S**).


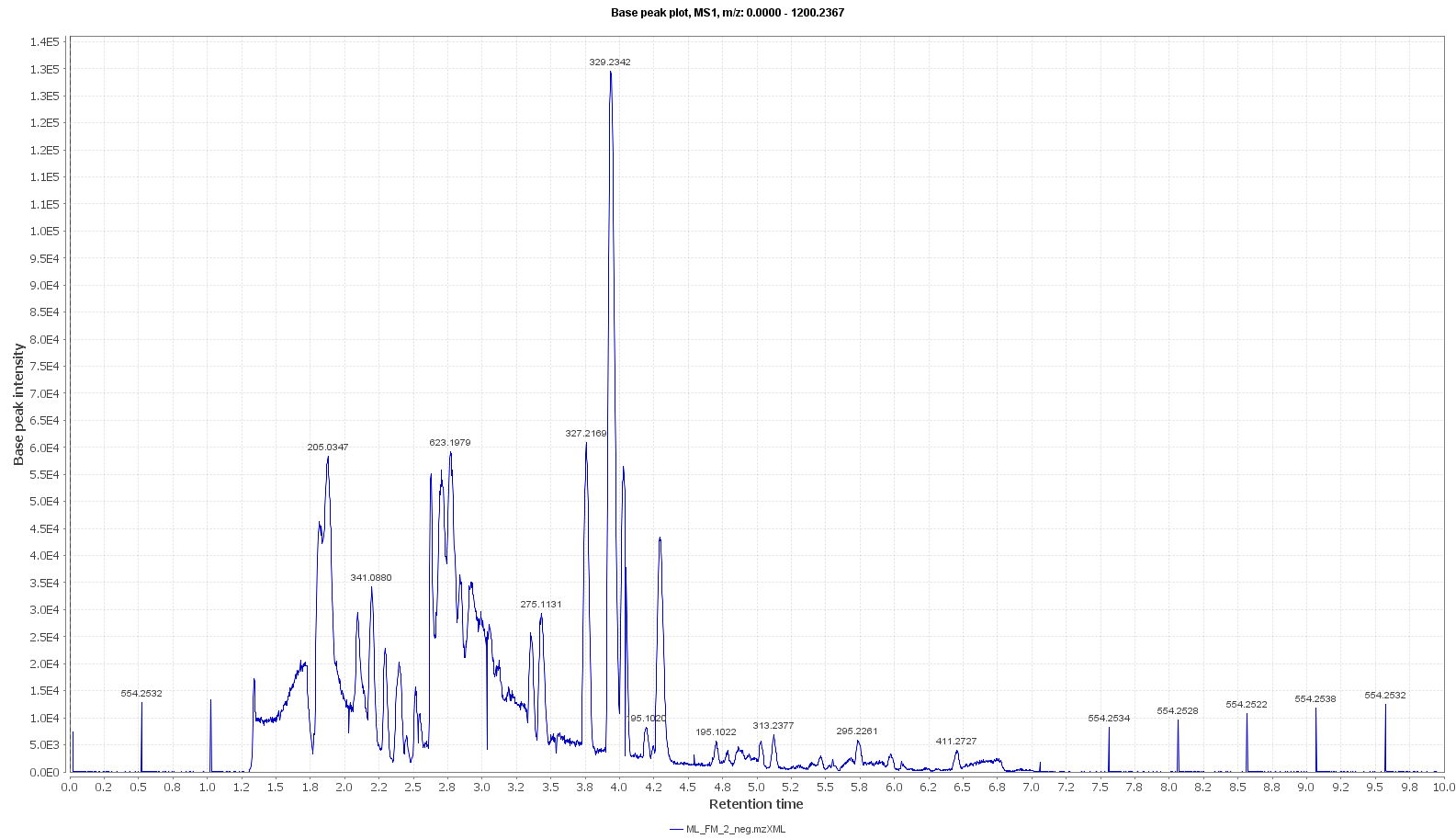
**Figure (S4):** LC/HRMS-negative mode total ion chromatogram of ISP2 solid extract (**ISP2-S**).


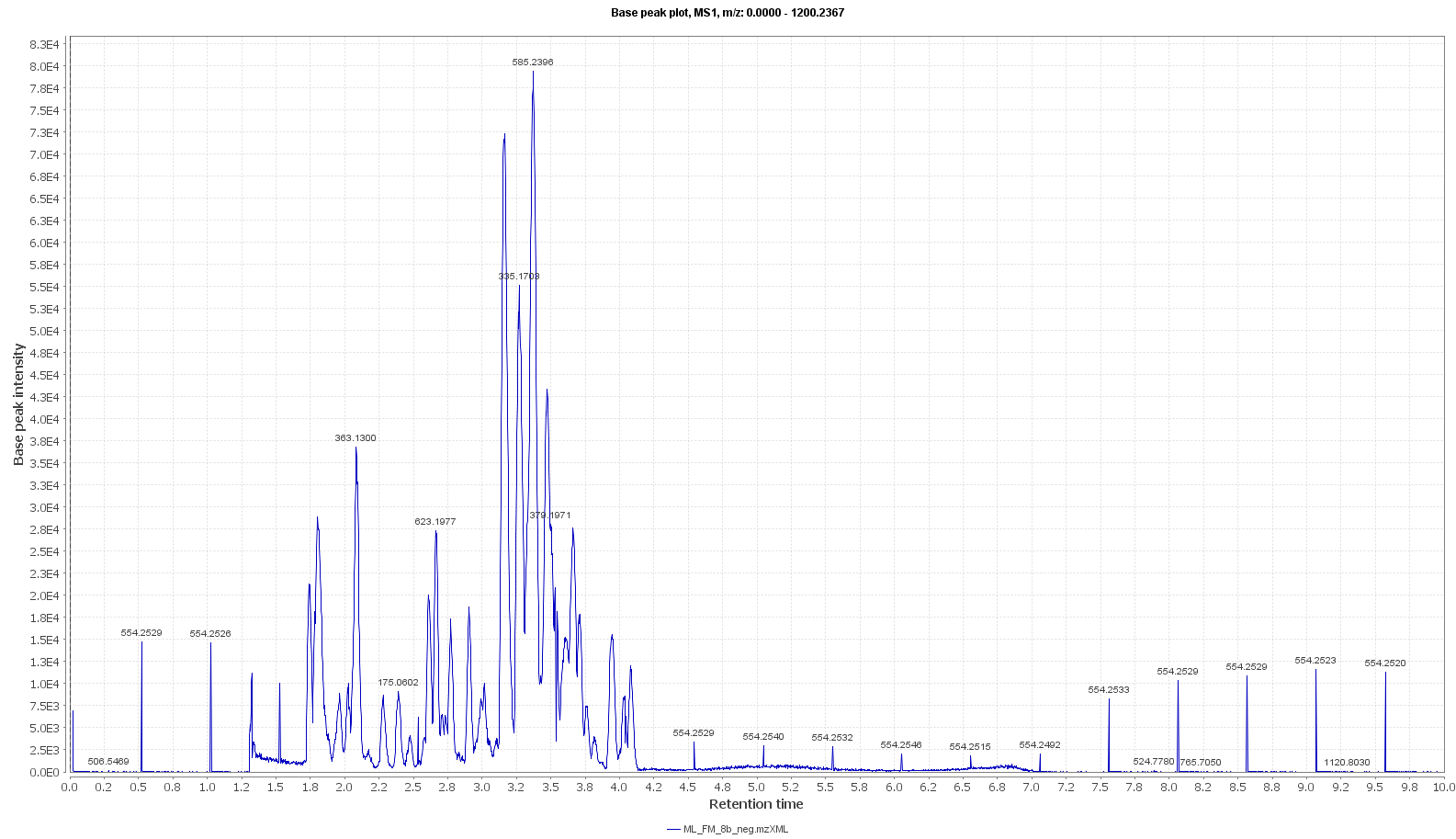
**Figure (S5):** LC/HRMS-negative mode total ion chromatogram of Oligo solid extract (**Oligo-S**).


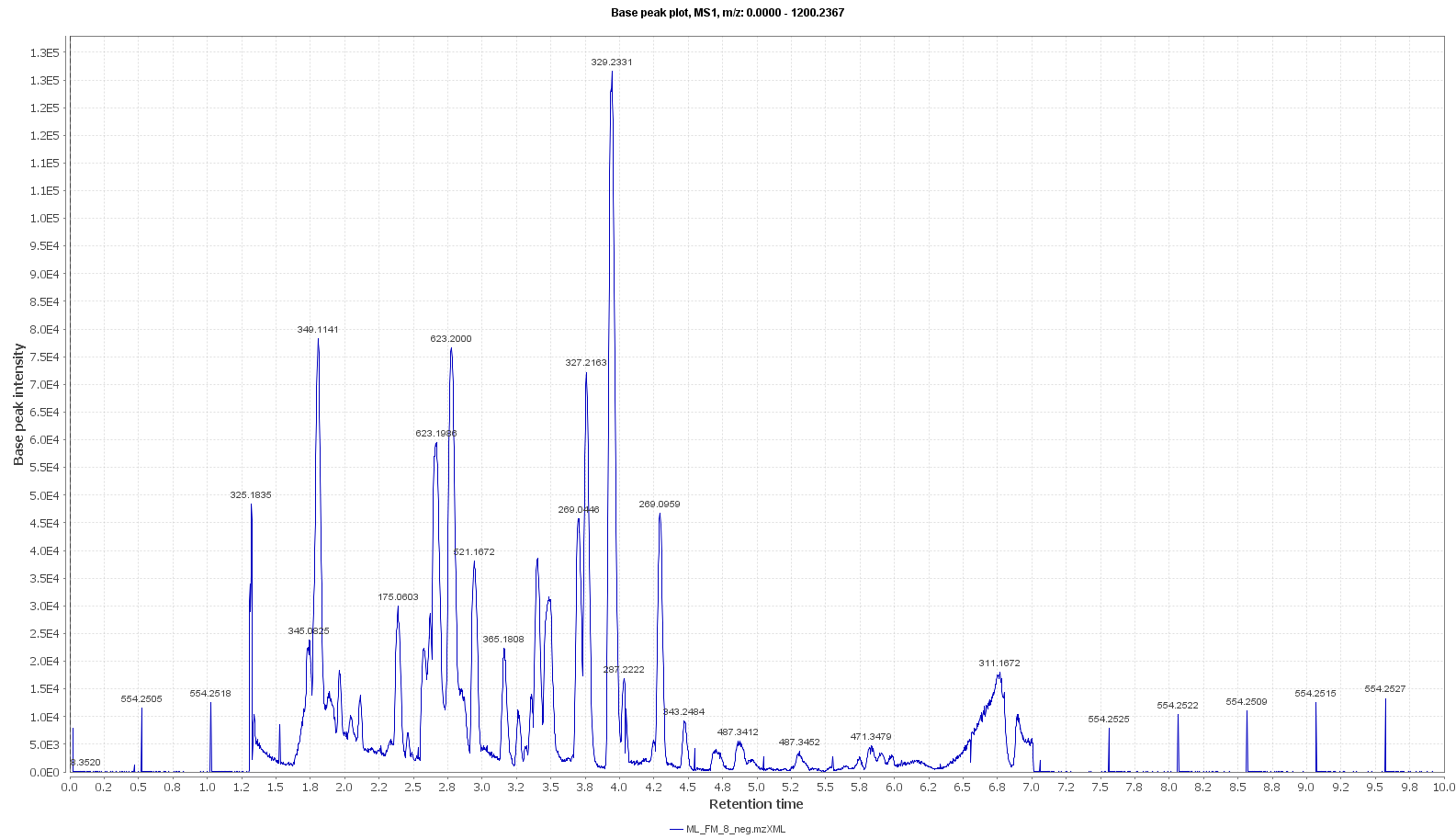
**Figure (S6)**: LC/HRMS-positive mode total ion chromatogram of Oligo solid extract (**Oligo-S**).


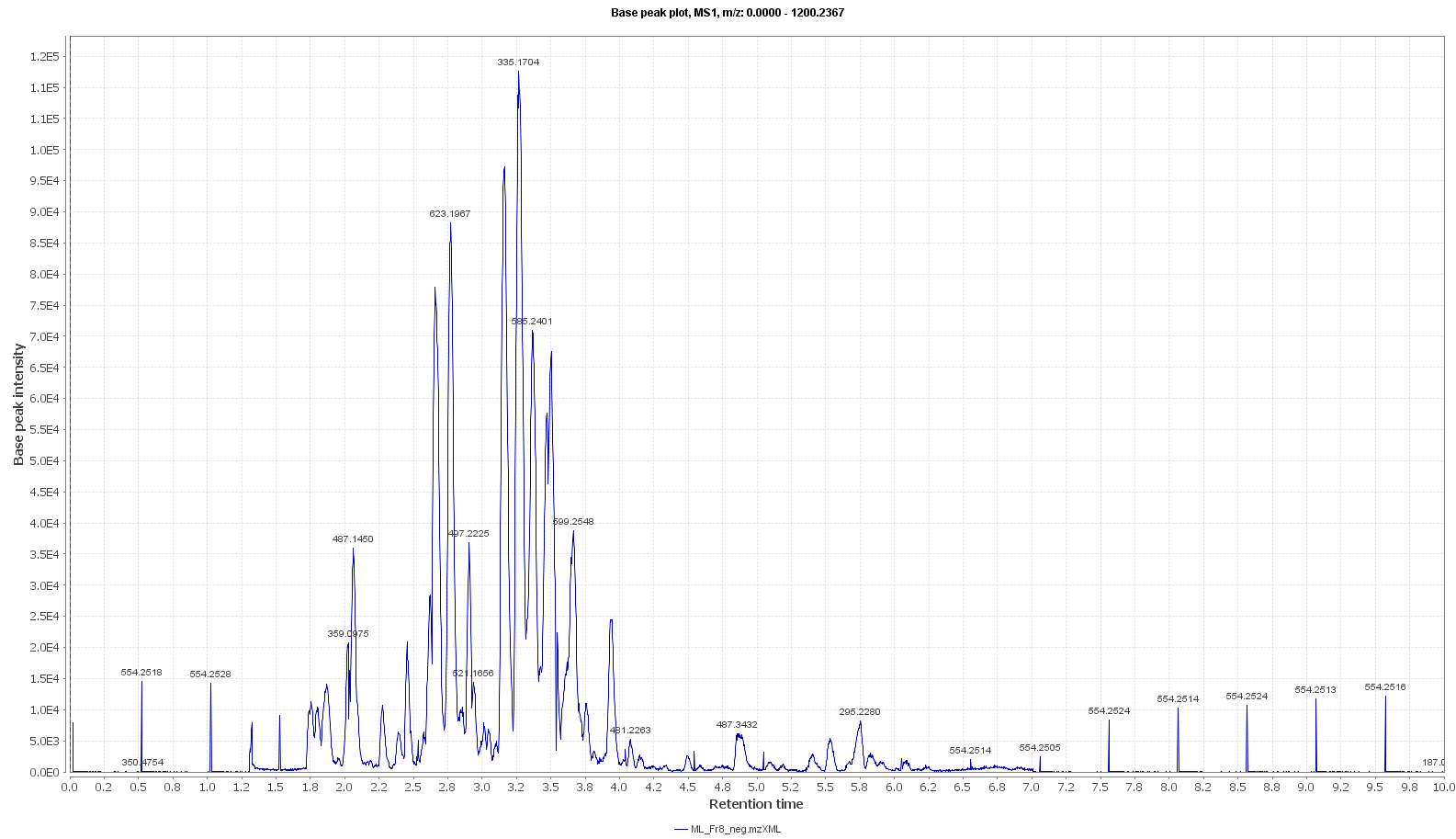
**Figure (S7):** LC/HRMS-negative mode total ion chromatogram of M1 liquid extract (**M1-L).**


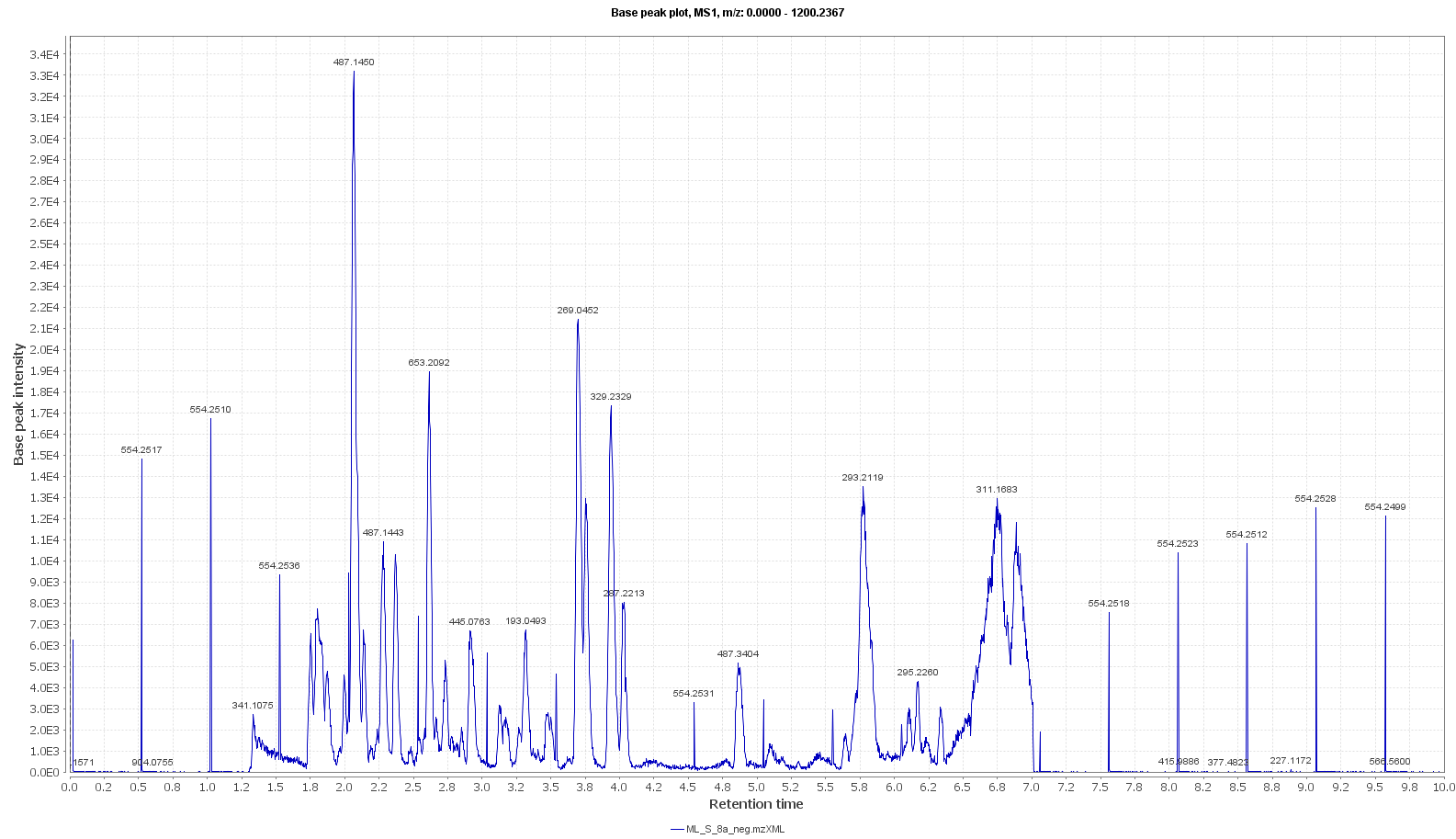
**Figure (S8):** LC/HRMS-positive mode total ion chromatogram of the M1 liquid extract (**M1-L).**


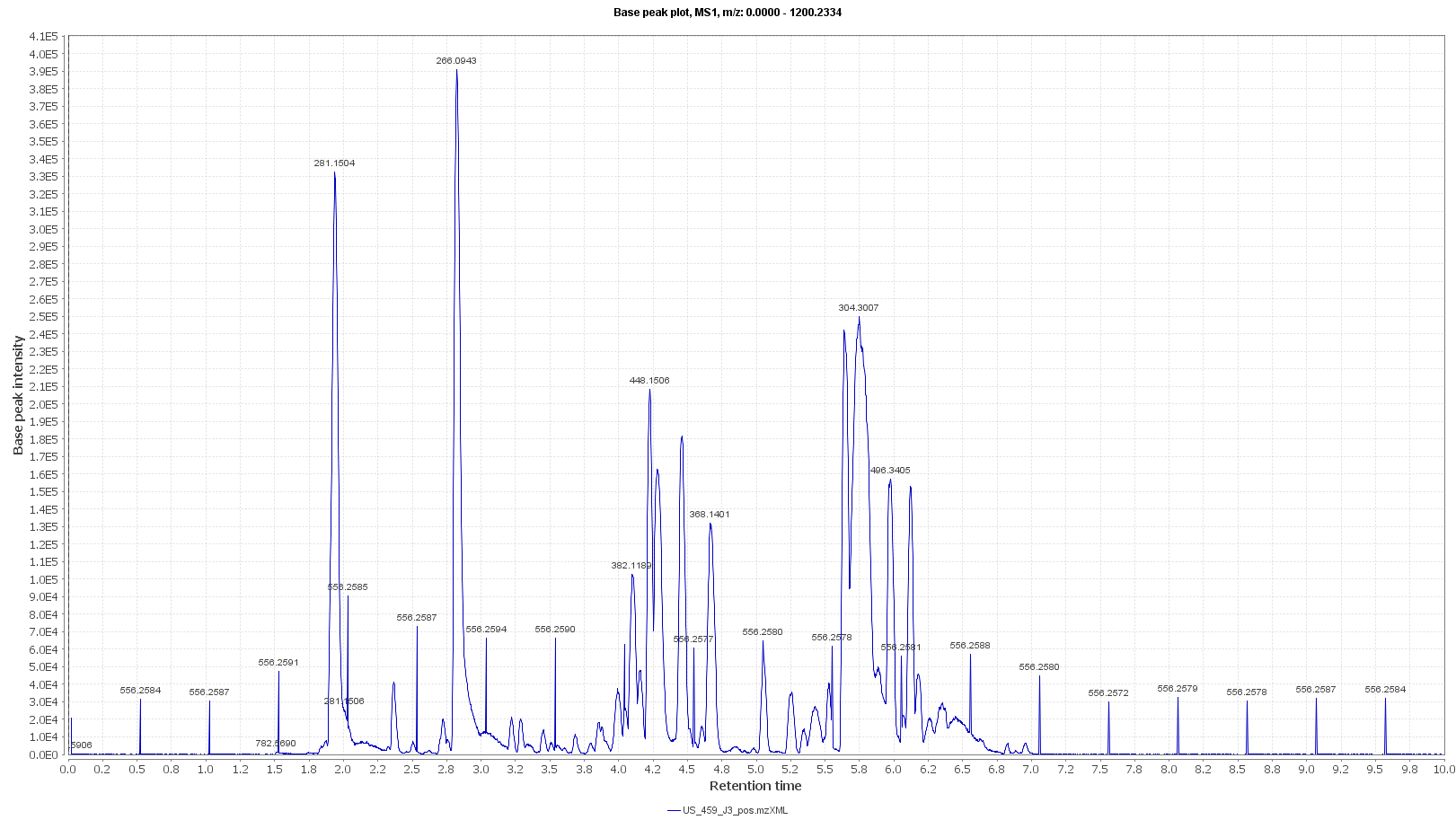
**Figure (S9):** LC/HRMS-negative mode total ion chromatogram of ISP2 liquid extract (**ISP2-L**).


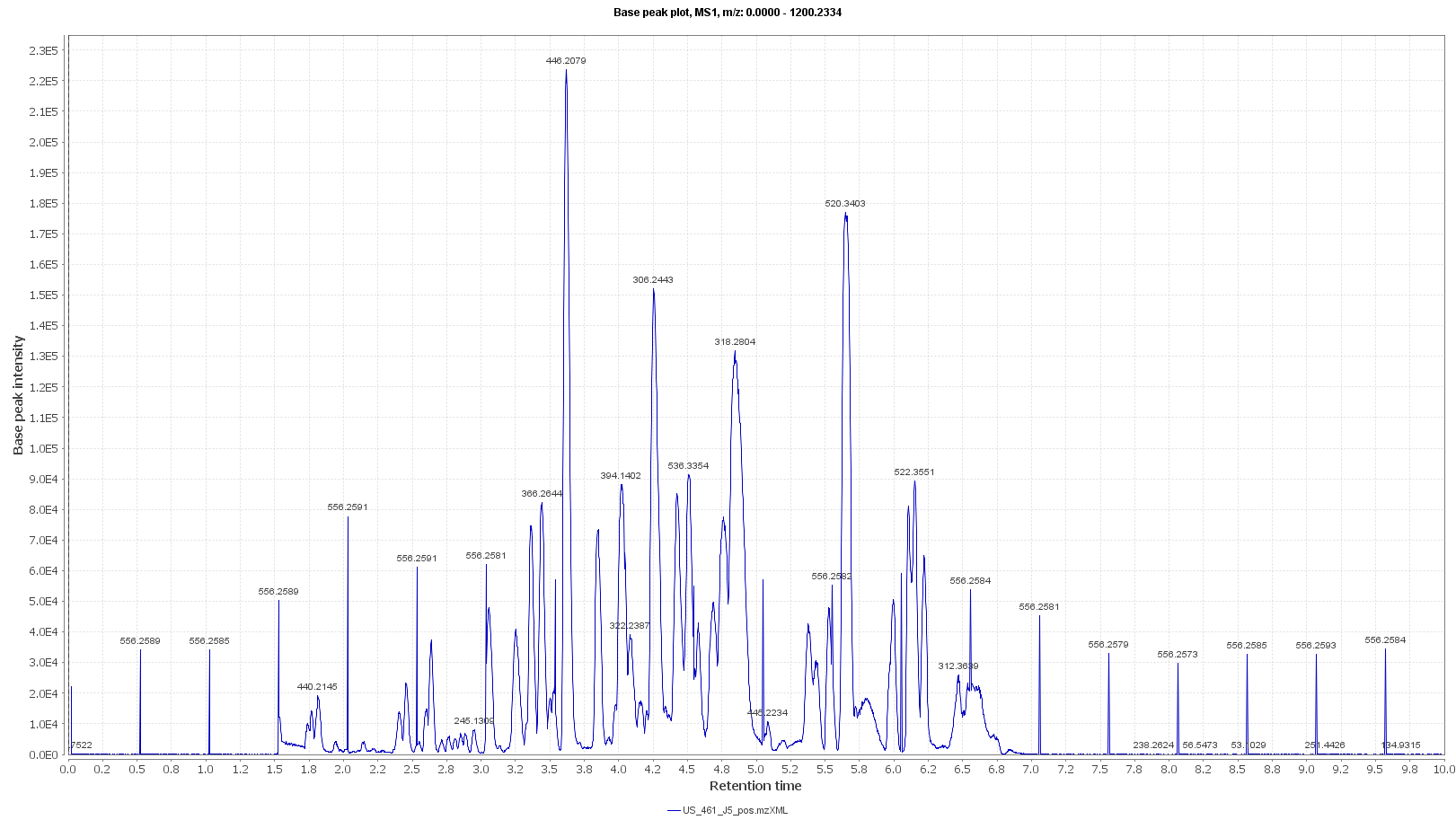
**Figure (S10):** LC/HRMS-positive mode total ion chromatogram of ISP2 liquid extract (**ISP2-L**).


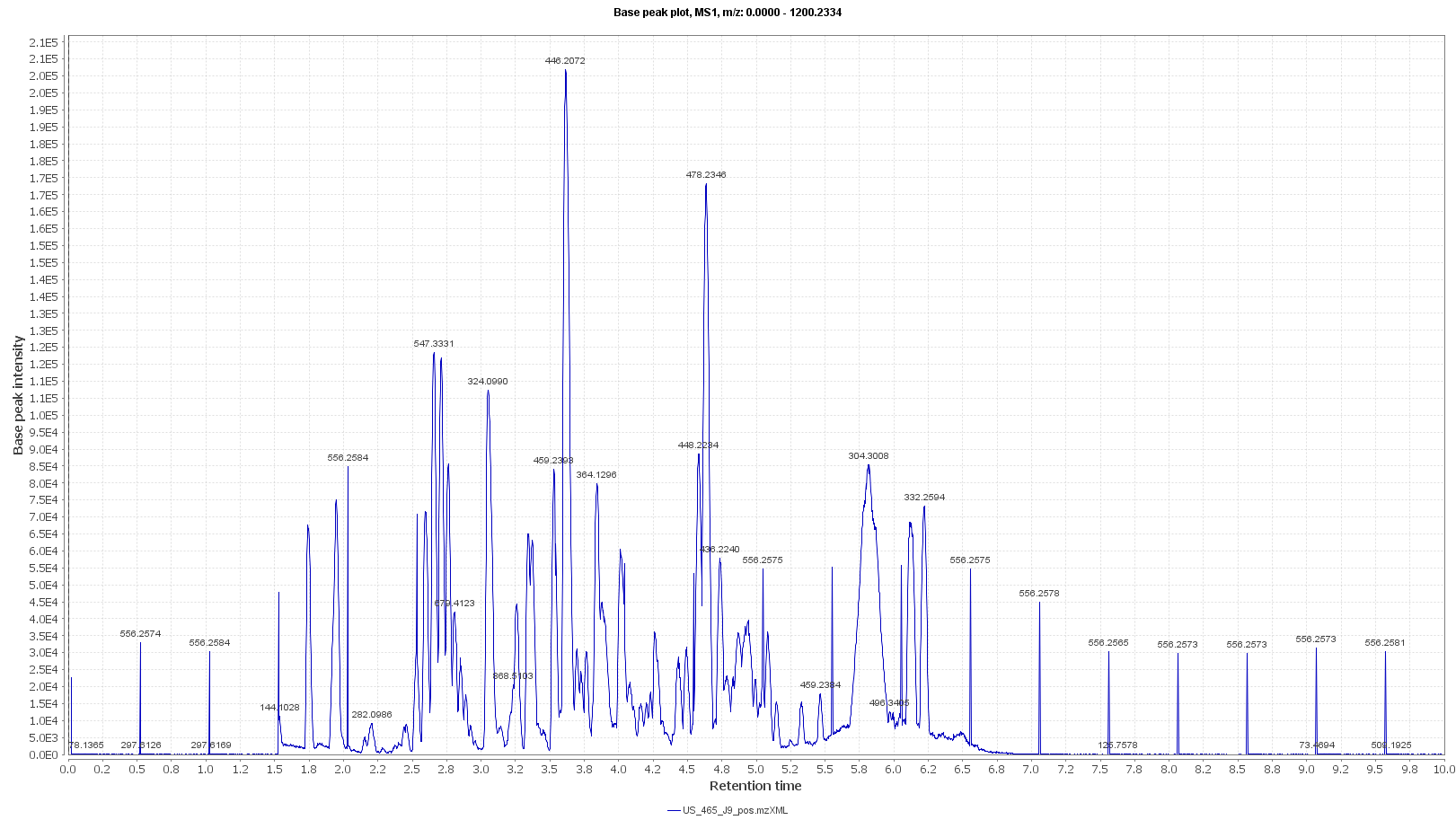


**Figure (S11):** LC/HRMS-positive mode total ion chromatogram of Oligo liquid extract (**Oligo-L**)


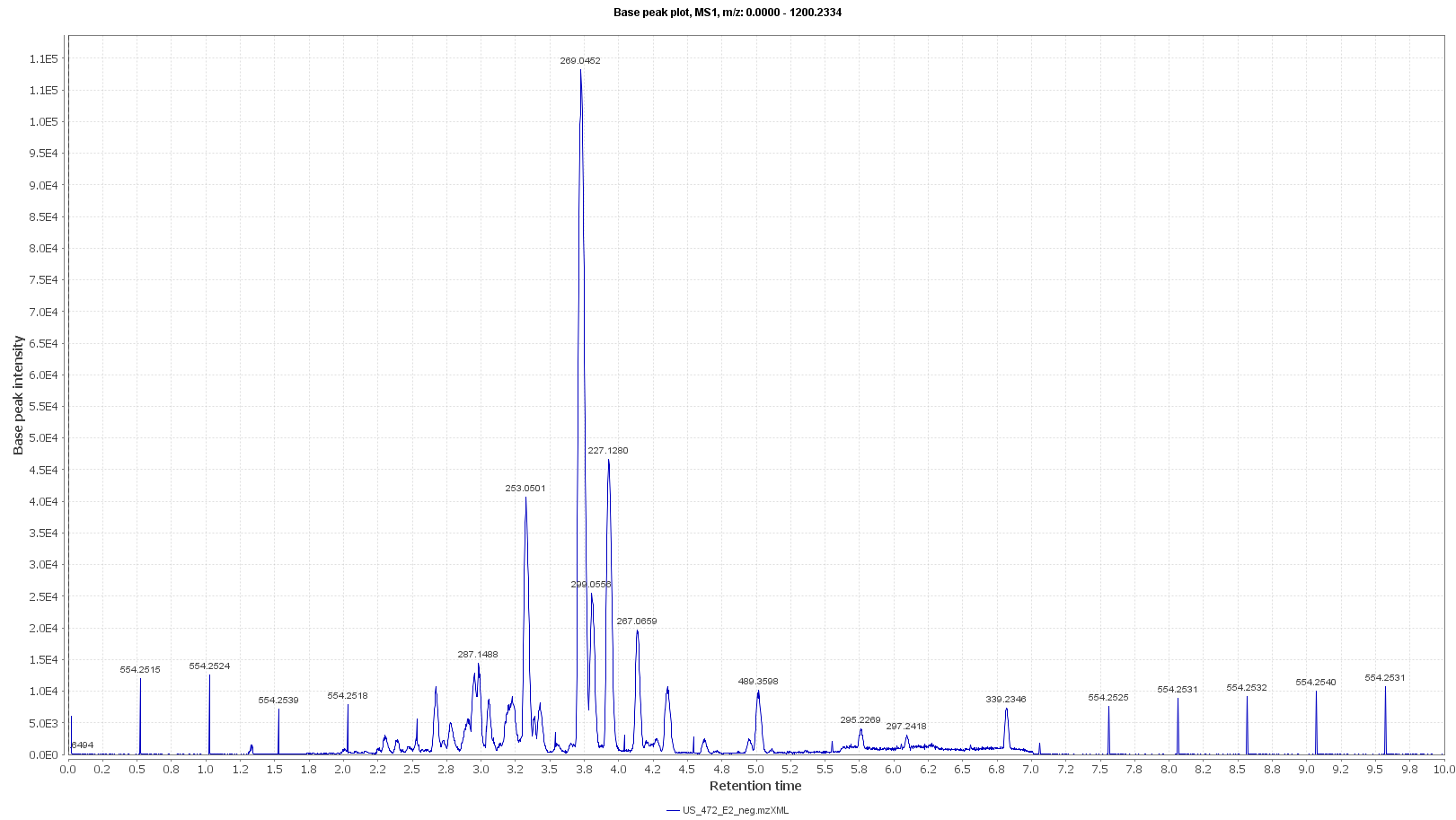


**Figure (S12):** LC/HRMS-negative mode total ion chromatogram of Oligo liquid extract (**Oligo-L**).

- **Media composition:**

1. **ISP-2 media: [**4 g Yeast Extract, 10 g Malt Extract, 4 g Dextrose, 1L Distilled water and 20 g agar**]** [1].
2. **Oligotrophic media (OLIGO)**: [0.5 g tryptone, 0.1 g sodium glycerophosphate, 0.05 g yeast extract, 1 L filtered seawater, 12 g Noble agar] [2].
3. **M1 media:** [10 g of starch, 4 g of yeast extract, 2 g of peptone, 18 g of agar, and 1L of natural seawater] [3].

- **Mzmine data preprocessing parameters:**

Using ProteoWizard's MassConvert programme, the raw data were initially divided into two data sets based on the ionization method. The sliced data sets were added to Mzmine 2.20, a programme for analyzing mass spectrometry data differentially. Mass adducts, fragments, and complexes were predominantly detected and sorted using Mzmine 2.20, and the high-resolution mass spectrum (MS^1^) data set was deconvoluted and deisotoped. The spectrum was cropped to the range of 2 to 35 minutes. Using the chromatogram builder, the peaks in the samples and blanks were found. With a centroid detector threshold greater than the noise level set to 1.0E4 and an MS level of 1, mass ion peaks were identified. The chromatogram builder was then utilized with the minimum time span set to 0.2 minutes, the minimum height set to 1.0E4, and the minimum m/z tolerance set to 0.001 m/z or 5.0 ppm, respectively. After that, chromatogram deconvolution was used to identify the distinct peaks. The local minimum search algorithm was used, which had the following parameters: chromatographic threshold of 90%, search minimum in RT range of 0.4 min, minimum relative height of 5%, minimum absolute height of 3.0E4, minimum ratio of peak top/edge of 2, and peak duration range of 0.3-5 min. Additionally, isotopes were found by utilizing the isotopic peaks grouper (m/z tolerance: 0.001 m/z or 5.0 ppm, retention time tolerance: 0.2 absolute (min), maximum charge: 2, and representative isotope: most intense). Reduced inter-batch variation was achieved by using the retention time normalizer (m/z tolerance: 0.001 m/z or 5.0 ppm, retention time tolerance: 5.0 absolute (min), and minimum standard intensity: 5.0E3). The join aligner's parameters, which were m/z tolerance of 0.001 m/z or 5.0 ppm, weight for m/z of 20, and retention time tolerance of 5.0 relative (%), were used to align all of the peak lists. With a 1.0% intensity tolerance, a 0.001 m/z or 5.0 ppm m/z tolerance, and a 0.5 absolute (min) retention time tolerance, the gap filling peak finder was used to identify missing peaks. The following adducts were investigated: Na-H, K-H, NH4, formate, and ACN + H (maximum relative adduct peak height: 30%), with RT tolerance of 0.2 absolute (min), 0.001 m/z, and 5.0 ppm. In addition, a complex search was conducted using the following parameters: [M + H]+ for ESI positive mode, [M−H^−^] for ESI negative mode, retention time tolerance of 0.2 absolute (min), m/z tolerance of 0.001 m/z or 5.0 ppm, and a maximum complex peak height of 50%. After processing, the data set was put through peak identification and molecular formula prediction. The molecular formula data set from Antibase® (February 2012) and Marinlit® (September 2012), from which the monoisotopic precise masses were computed, was used to generate an established library using an algorithm. Instead of using the manually curated Antibase and MarinLit databases, which do not distinguish between monoisotopic, average, and most abundant mass, the standard library was used. As a bespoke database for peak identification and dereplication, the developed library was then connected to Mzmine[4].

1. Shirling, E.B.; Gottlieb, D. Methods For Characterization of Streptomyces Species. *Int. J. Syst. Bacteriol.* **1966**, *16*, 313–314.

2. Olson, J.B.; Lord, C.C.; McCarthy, P.J. Improved Recoverability of Microbial Colonies from Marine Sponge Samples. *Microb. Ecol.* **2000**, *40*, 139–147, doi:10.1007/s002480000058.

3. Mincer, T.J.; Jensen, P.R.; Kauffman, C.A.; Fenical, W. Widespread and Persistent Populations of a Major New Marine Actinomycete Taxon in Ocean Sediments. *Appl. Environ. Microbiol.* **2002**, *68*, 5005–5011.

4. Abdelmohsen, U.R.; Cheng, C.; Viegelmann, C.; Zhang, T.; Grkovic, T.; Ahmed, S.; Quinn, R.J.; Hentschel, U.; Edrada-ebel, R. Dereplication Strategies for Targeted Isolation of New Antitrypanosomal Actinosporins A and B from a Marine Sponge Associated-Actinokineospora Sp. EG49. *Mar. Drugs* **2014**, *12*, 1220–1244, doi:10.3390/md12031220.
